# MicroRNA-144 is regulated by CP2 and decreases *COX-2* expression and PGE2 production in mouse ovarian granulosa cells

**DOI:** 10.1038/cddis.2017.24

**Published:** 2017-02-09

**Authors:** Jiawei Zhou, Bin Lei, Huanan Li, Lihua Zhu, Lei Wang, Hu Tao, Shuqi Mei, Fenge Li

**Affiliations:** 1 Key Laboratory of Pig Genetics and Breeding of Ministry of Agriculture & Key Laboratory of Agricultural Animal Genetics, Breeding and Reproduction of Ministry of Education, Huazhong Agricultural University, Wuhan, China; 2 Hubei Key Laboratory of Animal Embryo Engineering and Molecular Breeding, Hubei Academy of Agriculture Science, Wuhan, China; 3 The Cooperative Innovation Centre for Sustainable Pig Production, Wuhan, China

## Abstract

Mammalian folliculogenesis is a complex process in which primordial follicles develop into pre-ovulatory follicles, followed by ovulation to release mature oocytes. In this study, we explored the role of miR-144 in ovulation. miR-144 was one of the differentially expressed microRNAs, which showed 5.59-fold changes, in pre-ovulatory ovarian follicles between Large White and Chinese Taihu sows detected by Solexa deep sequencing. We demonstrated that overexpression of miR-144 significantly decreased the luciferase reporter activity under the control of the cyclooxygenase-2 (*COX-2*) or mothers against decapentaplegic homologue 4 (*Smad4*) 3'-untranslated region (3'-UTR) and suppressed *COX-2* and *Smad4* expression. In contrast, a miR-144 inhibitor increased *COX-2* and *Smad4* expression in mouse granulosa cells (mGCs). Meanwhile, *Smad4* upregulated *COX-2* expression, but this effect was abolished when the mGCs were treated with the transforming growth factor beta signalling pathway inhibitor SB431542. Moreover, luciferase reporter, chromatin immunoprecipitation and electrophoretic mobility shift assay results showed that the transcription factor CP2 upregulated miR-144 expression, which partially contributed to the suppression of *COX-2* in mGCs. Both CP2 and miR-144 alter prostaglandin E2 (PGE2) production by regulating *COX-2* expression. In addition, miR-144 regulated mGC apoptosis and affected follicular atresia, but these activities did not appear to be through *COX-2* and *Smad4*. Taken together, we revealed an important CP2/miR-144/*COX-2*/PGE2/ovulation pathway in mGCs.

Chinese Taihu (CT) pigs farrow three to five more piglets per litter than American or European pig breeds, which has been intensely studied.^1^ The greater litter size at farrowing in multiparous CT sows is partly due to a greater ovulation rate, increased embryonic survival, a greater uterine capacity and a lower fertilization failure rate.^2, 3^ A better understanding of follicular growth and ovulation is essential for enhancing the fertilization rate and minimizing early embryonic losses in pigs.

In the mammalian ovary, folliculogenesis begins from primordial germ cells, and proceeds through the primary follicle stage to the mature follicle stage and finally produces a pre-ovulatory follicle and releases an oocyte in response to a surge in luteinizing hormone (LH). A few follicles develop to ovulation, and >99% of ovarian follicles will be lost as a result of atresia.^4, 5^ The complexity of folliculogenesis indicates that tightly regulated gene expression and multiple gene interactions are required for successful ovulation. Several endocrine and intra-ovarian factors are related to ovulation.^6, 7, 8^ Among these, prostaglandin E2 (PGE2) is a critical mediator of ovulation.^9^

Cyclooxygenase (*COX*) is the key enzyme that controls the rate-limiting step of prostaglandin synthesis.^10^ To date, two well-characterized isoforms of *COX* have been identified, *COX-1* and *COX-2*. *COX-1* is a constitutive enzyme, whereas *COX-2* is undetectable in a variety of cell types and can be a significantly induced by various hormones, growth factors and cytokines.^10^ In the monkey, *COX-1* is not detected in the granulosa cells of large antral follicles. However, *COX-2* expression substantially increased in granulosa cells of pre-ovulatory ovarian follicles stimulated by human chorionic gonadotropin (hCG).^11^ A large number of studies have shown that inhibition of *COX-2* resulted in ovulation failure.^12, 13^ Moreover, mice deficient in *COX-1* are fertile, whereas *COX-2*-deficient mice exhibit multiple failures in female reproductive processes, including ovulation, fertilization, implantation and decidualization.^14^ Furthermore, ovulation is restored in the *COX-2*-deficient mice by simultaneous treatment with PGE2.^15^ Overall, these studies strongly suggest that *COX-2*-derived PGE2 is crucial for ovulation.

MicroRNAs (miRNAs) are short, non-coding RNAs, 19–22 nucleotides in length, which act by targeting partially complementary sequences within the mRNA 3′-untranslated region (3′-UTR), leading to functional repression of target transcripts. These molecules are involved in multiple biological processes.^16^ Many studies have shown that miRNAs have important roles in follicular development, follicular atresia, hormone modulation, cell apoptosis and cell proliferation.^17, 18, 19, 20^ However, the role of miRNAs in ovulation is unclear thus far.

In this study, we found that the expression of miR-144 was significantly different between pre-ovulatory ovarian follicles of Large White (LW) and Taihu sows. Both *in vivo* and *in vitro* analyses demonstrated that transcription factor CP2 bound to and activated the miR-144 promoter in mouse granulosa cells (mGCs). Luciferase activity and expression level analyses showed that *COX-2* and *Smad4* were targets of miR-144. *Smad4* regulated *COX-2* levels via the transforming growth factor beta (TGF-*β*) signalling pathway. Our research indicates that miR-144 is involved in ovulation by suppressing PGE2 production in mammals.

## Results

### miR-144 was differentially expressed in CT and LW sows

To explore the mechanism of ovulation, we investigated whether specific miRNAs displayed breed-modulated expression in the ovarian follicles. Two independent small RNA libraries from pre-ovulatory ovarian follicles of CT and LW sows were sequenced with the high-throughput Illumina Solexa system (Beijing Genomics Institute, Shenzhen, China). In CT and LW sows, 679 and 713 known miRNAs were detected, respectively. Three hundred ninety miRNAs were differentially expressed between the two breeds, with 124 miRNAs upregulated and 266 miRNAs downregulated in CT sows (*P*<0.05, |Fold change|>1) (Supplementary Table S1). Quantitative real-time PCR (qRT-PCR) analyses showed that the expression profiles of seven miRNAs (let-7a, miR-125a, miR-144, miR-2423, miR-3613-5p, miR-331* and miR-4028-3p) were consistent with the results from deep sequencing (Supplementary Figure S1).

### miR-144 negatively regulates *COX-2* in mGCs

**Figure 1:**
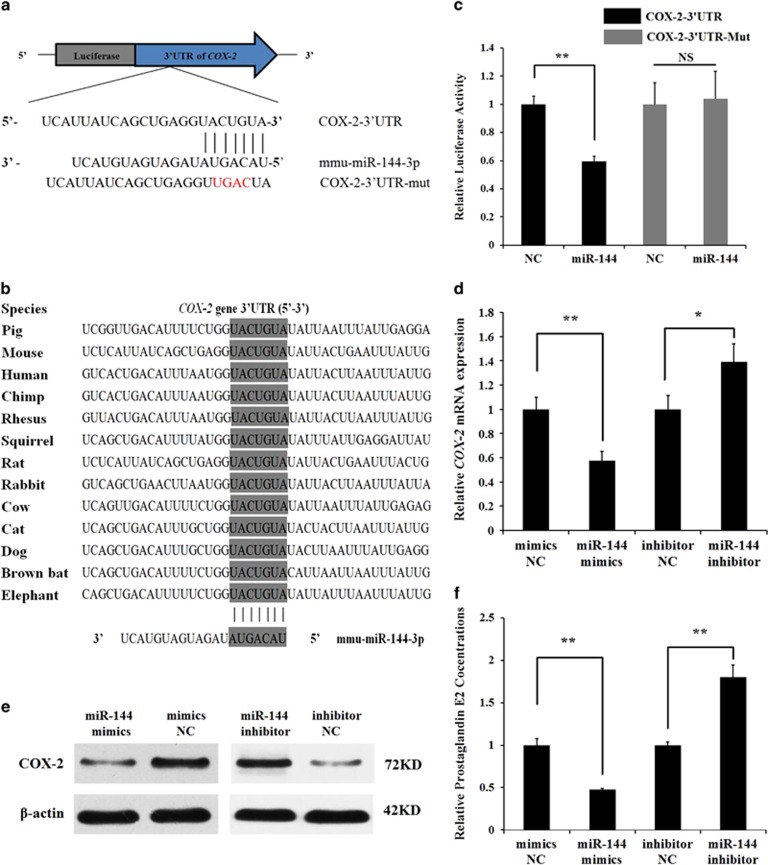
Identification of *COX-2* as a direct target of miR-144 in mGCs. (**a**) Binding sites for miR-144 in the 3′-UTR of *COX-2* predicted by TargetScan. Red font indicates sequences that were mutated to abolish the interaction between miR-144 and the *COX-2* 3′-UTR. (**b**) The miR-144-binding site sequences in the *COX-2* 3′-UTR in different species. (**c**) Luciferase activity was analyzed 24 h after CHO-K1 cells were co-transfected with *pmiRGLO-COX-2-3'-UTR* or *pmiRGLO-COX-2-3'-UTR-Mut* and miR-144 mimics or mimics NC. (**d**) Endogenous *COX-2* mRNA levels were detected 48 h after mGCs were transfected with miR-144 mimics, mimics NC, miR-144 inhibitor or inhibitor NC. (**e**) Western blot analysis was used to detect endogenous COX-2 protein expression level 72 h after mGCs were transfected with miR-144 mimics, mimics NC, miR-144 inhibitor or inhibitor NC. (**f**) ELISA was used to detect endogenous PGE2 production 48 h after mGCs were transfected with miR-144 mimics, mimics NC, miR-144 inhibitor or inhibitor NC. The results are expressed as the mean±S.E.M. (three independent replicates per group). *P<0.05, **P<0.01, N.S.=nonsignificant

miR-144 was one of the differentially expressed miRNAs and showed a 5.59-fold change in pre-ovulatory ovarian follicles between LW and CT sows with Solexa deep sequencing technology. Therefore, miR-144 was selected as a candidate miRNA for analysis of ovulation. TargetScan and RNAhybrid were used to detect potential target genes of miR-144. *COX-2* was predicted to be a target of miR-144. In addition, the miR-144-binding seed sequences in the *COX-2* 3′-UTR were highly conserved in mammals (Figures 1a and b).

The dual-luciferase reporter system was used to analyze the interaction between miR-144 and the *COX-2* gene. We co-transfected miR-144 mimics and a luciferase reporter vector containing the mouse 233 bp *COX-2-3*′*-UTR* (*pmirGLO-COX-2-3*′-*UTR*) into Chinese hamster ovarian (CHO-K1) cells, and luciferase activity was significantly suppressed. However, luciferase activity was unchanged when we co-transfected miR-144 mimics and *pmirGLO-COX-2-3*′*-UTR-Mut* into mGCs (Figure 1c). qRT-PCR and western blot analyses revealed that *COX-2* mRNA and protein expression levels were significantly suppressed after miR-144 mimics was transfected into mGCs and pig kidney (PK-15) cells, whereas inhibition of miR-144 increased *COX-2* mRNA and protein in mGCs and PK-15 cells (Figures 1d, e and Supplementary Figure S2).

PGE2 production has a key role in the regulation of ovulation.^12, 15^ Thus, it is essential to determine whether miR-144 affects PGE2 production in mGCs by regulating *COX-2* expression. *COX-2* expression levels were significantly increased and suppressed after *pcDNA3.1-COX-2* (pc-COX-2) or siRNA-COX-2 (si-COX-2) was transfected into mGCs, respectively (Supplementary Figures S3a-d). As shown in Supplementary Figure S3e, *COX-2* upregulated PGE2 production. Interestingly, miR-144 suppressed PGE2 production by reducing the expression of *COX-2* (Figure 1f). These results show that miR-144 affects ovulation by regulating *COX-2* expression level and PGE2 production in mGCs.

### miR-144 directly targets the *Smad4* gene in mGCs

**Figure 2:**
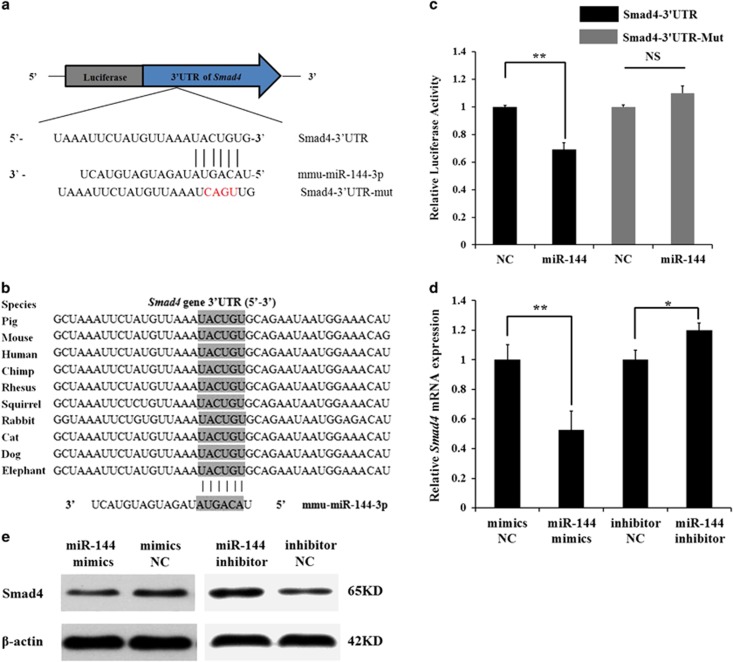
Identification of *Smad4* as a direct target of miR-144 in mGCs. (**a**) Binding sites for miR-144 in the 3′-UTR of *Smad4* predicted by TargetScan. Red font indicates sequences that were mutated to abolish the interaction between miR-144 and the *Smad4* 3′-UTR. (**b**) The miR-144-binding site sequences in the *Smad4* 3′-UTR in different species. (**c**) Luciferase activity was analyzed 24 h after CHO-K1 cells were co-transfected with *pmiRGLO-Smad4-3*′*-UTR* or *pmiRGLO-Smad4-3*′*-UTR-Mut* and miR-144 mimics or mimics NC. (**d**) Endogenous *Smad4* mRNA levels were detected 48 h after mGCs were transfected with miR-144 mimics, mimics NC, miR-144 inhibitor or inhibitor NC. (**e**) Western blot analysis was used to detect endogenous Smad4 protein expression level 72 h after mGCs were transfected with miR-144 mimics, mimics NC, miR-144 inhibitor or inhibitor NC. The results are expressed as the mean±S.E.M. (three independent replicates per group). *P<0.05, **P<0.01, N.S.=nonsignificant

Increasing evidence has indicated that many members of the TGF-*β* superfamily have important roles in follicular development and ovulation.^21^ Here, we predicted that *Smad4*, a key gene in the TGF-*β* pathway, may be a target of miR-144. The miR-144-binding seed sequences in the *Smad4* 3′-UTR were also highly conserved in mammals (Figures 2a and b). A luciferase reporter analysis was used to determine the binding sites of miR-144 in the *Smad4* 3′-UTR. The *pmirGLO-Smad4-3*′*-UTR* luciferase reporter was co-transfected with miR-144 mimics or mimics negative control (NC) into CHO-K1 cells, and luciferase activity was significantly suppressed by miR-144. Meanwhile, miR-144 had no effect on a *Smad4* 3′-UTR mutated dual-luciferase construct (Figure 2c). qRT-PCR and western blot analysis revealed that miR-144 significantly inhibited the *Smad4* mRNA level and protein level (Figures 2d and e). These results confirm that miR-144 regulates the TGF-*β* signalling pathway via *Smad4* in mGCs.

### *Smad4* regulates *COX-2* expression through the TGF-*β* signalling pathway in mGCs

**Figure 3:**
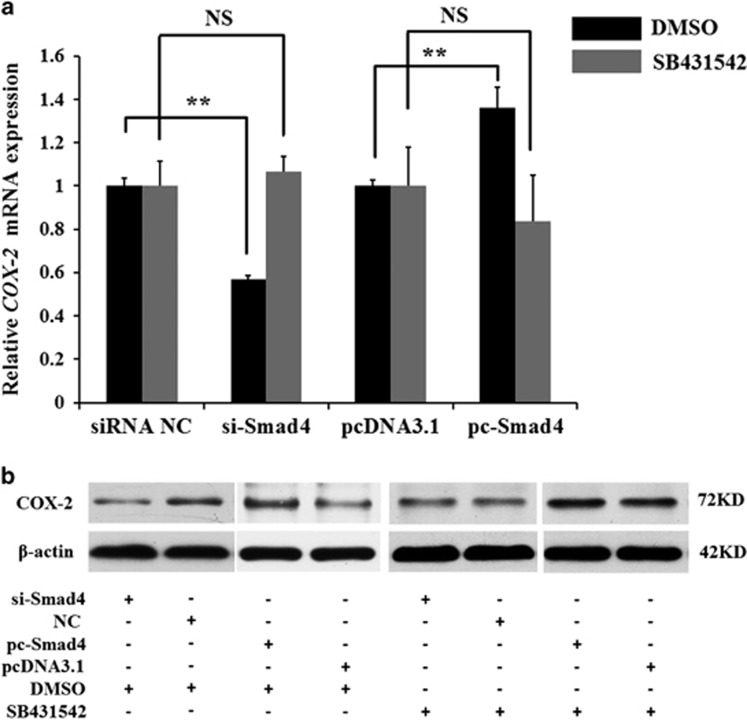
*Smad4* promotes the expression of the *COX-2* gene via the TGF-*β* signalling pathway in mGCs. Mouse GCs were pretreated with DMSO or 5 *μ*M SB431542 (prepared in DMSO) for 30 min and then transfected with siRNA-Smad4, NC, and *pcDNA3.1-Smad4* or *pcDNA3.1*. (**a**) qRT-PCR was used to detect endogenous *COX-2* mRNA 48 h after transfection. (**b**) Western blot analysis was used to detect COX-2 protein expression levels 72 h after transfection. The results are expressed as the mean±S.E.M. (three independent replicates per group). **P<0.01, N.S.=nonsignificant

Previous studies have shown that the TGF-*β* signalling pathway regulates *COX-2* expression.^22^ Therefore, *Smad4* may be involved in regulating *COX-2* expression. Transfection of siRNA-Smad4 (si-Smad4) into mGCs knocked down *Smad4* expression (Supplementary Figures S4a and b), which resulted in significantly decreased *COX-2* expression (Figures 3a and b). *pcDNA3.1-Smad4* (pc-Smad4) was transfected into mGCs and substantially increased *Smad4* expression (Supplementary Figures S4c and d), followed by increased *COX-2* expression (Figures 3a and b). However, the effect of *Smad4* on *COX-2* expression disappeared when mGCs were treated with SB431542 (a TGF-*β* signalling pathway inhibitor) (Figures 3a and b). These results suggest that *Smad4* regulates *COX-2* expression via the TGF-*β* signalling pathway.

### Identification of the promoter region and regulatory elements of mouse miR-144

**Figure 4:**
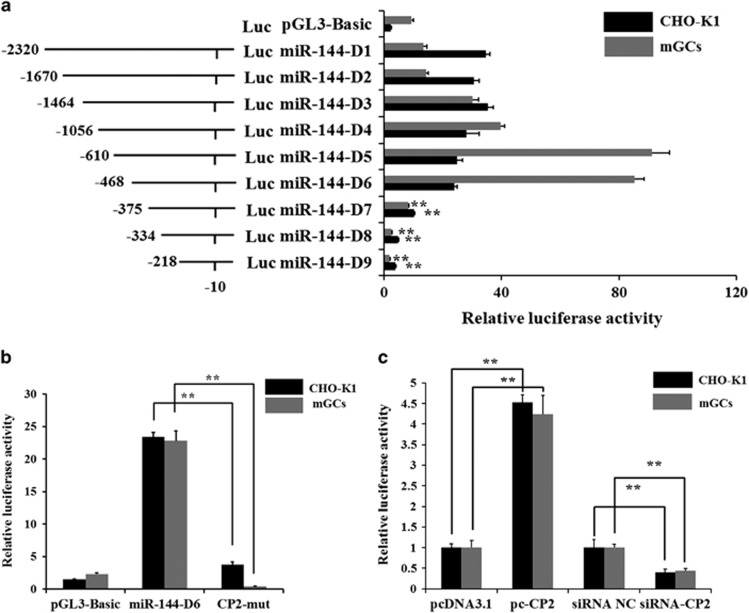
Identification of the CP2-binding site in the promoter regions of miR-144. (**a**) Luciferase assays show the activities of a series of deletion constructs in both mGCs and CHO-K1 cells. The left panel shows each deleted mutant linked with the luciferase gene in the *pGL3-basic* vector. The right panel indicates the relative activities of these deletion constructs. Luciferase activity was analyzed 24 h after transfection. (**b**) Point mutations in the CP2-binding sites of the miR-144 promoter were analyzed using luciferase assays. Luciferase activity was analyzed 24 h after transfection. (**c**) Luciferase activity was analyzed 24 h after mGCs and CHO-K1 cells were co-transfected with miR-144-D6 and *pcDNA3.1-CP2*, *pcDNA3.1*, siRNA-CP2 or siRNA NC. The results are expressed as the mean±S.E.M. (three independent replicates per group). **P<0.01

miR-144 was a negative regulator of *COX-2* expression, which prompted us to examine the transcriptional regulation of miR-144 in ovarian follicles. To identify the core promoter of mouse miR-144, a series of deletions of the mouse miR-144 potential promoter were used to drive luciferase gene expression, and luciferase activity was determined. Luciferase activity analysis in both mGCs and CHO-K1 cells revealed that *pGL3-miR-144-D6* (−468 bp to −375 bp) was required for miR-144 transcriptional activity (Figure 4a).

To further assess the transcription factors binding to the core promoter of miR-144, the CP2 transcription factor binding site was identified in the miR-144-D6 region using the transcription factor prediction software BIOBASE (Supplementary Figure S5a). In addition, sequence comparison analysis showed a highly conserved promoter in the CP2-binding site between mouse and pig (Supplementary Figure S5b). To confirm that CP2 can regulate the activity of the core promoter of mouse miR-144, site-directed mutagenesis was performed using the wild-type *pGL3-miR-144-D6* construct as a template. Luciferase activity analysis of both mGCs and CHO-K1 cells revealed that the mutated CP2-binding site (−411 bp to −402 bp) showed a substantial decrease in promoter activity (Figure 4b). *CP2* mRNA and protein expression levels were significantly increased or suppressed after *pcDNA3.1-CP2* (pc-CP2) or siRNA-CP2 (si-CP2) was transfected into mGCs, respectively (Supplementary Figures S6). The *pcDNA3.1-CP2*, *pcDNA3.1*, siRNA-CP2 or siRNA NC was co-transfected with the *pGL3-miR-144-D6* vector into mGCs. As shown in Figure 4c, overexpression of CP2 significantly increased miR-144 promoter activity. In contrast, knockdown of CP2 suppressed miR-144 promoter activity. These results indicate that the binding site of CP2 is important for miR-144 promoter activity.

### Transcription factor CP2 binds to the miR-144 promoter both *in vivo* and *in vitro*

**Figure 5:**
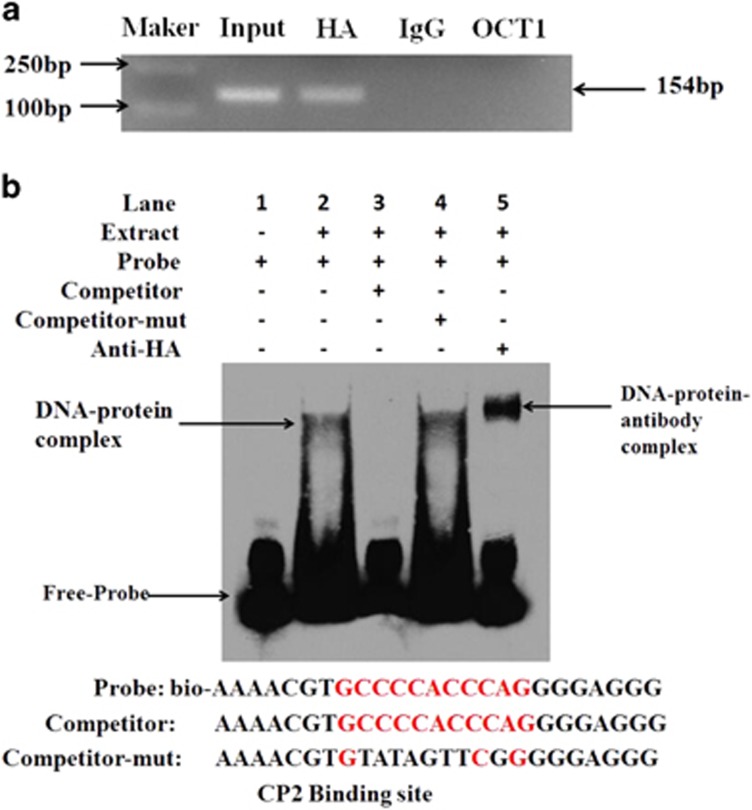
ChIP and EMSA showed that CP2 could bind to the miR-144 promoter *in vivo* and *in vitro*. (**a**) ChIP assay. The DNA fragments interacting with CP2 protein were pulled down by anti-HA antibodies. DNA isolated from immunoprecipitated material was amplified by PCR as the template. Total chromatin was used as the input. Normal mouse IgG and OCT1 were used as NCs. (**b**) EMSAs. The probes were incubated with nuclear extracts of mGCs in the absence or presence of a 50-fold excess of various competitor DNA oligos (mutant or unlabelled probes) or antibodies. The specific DNA–protein complex and DNA–protein–antibody complex bands are indicated by arrows. The sequences of the various probes are shown under the panel

Chromatin immunoprecipitation (ChIP) analysis was performed to investigate whether CP2 bound to the mouse miR-144 promoter *in vivo*. We constructed *pCMV-C-HA*-*CP2-CDS* and then transfected this vector into mGCs. A 154-bp DNA region was amplified from the anti-HA precipitates in mGCs, whereas DNA fragment was not amplified from the anti-IgG and anti-OCT1 antibody precipitates (Figure 5a). These results indicate that CP2 specifically binds to the mouse miR-144 promoter region *in vivo*.

Electrophoretic mobility shift assays (EMSAs) were used to further detect CP2 binding to the mouse miR-144 promoter *in vitro*. *pCMV-C-HA-CP2-CDS* was transfected into mGCs, and then, nuclear extracts were isolated from mGCs. A DNA–protein complex was detected when the CP2 probe (−419 bp to −395 bp) was incubated with nuclear extracts. An excess amount of unlabelled oligo DNA, but not the mutated CP2-binding site, could compete with this binding. Moreover, the supershift band appeared when the CP2 probe was incubated with nuclear extracts and anti-HA antibody (Figure 5b). These results suggest that CP2 binds to the mouse miR-144 promoter region *in vitro*.

### CP2 regulates the miR-144/451 cluster and *COX-2* expression in mGCs

**Figure 6:**
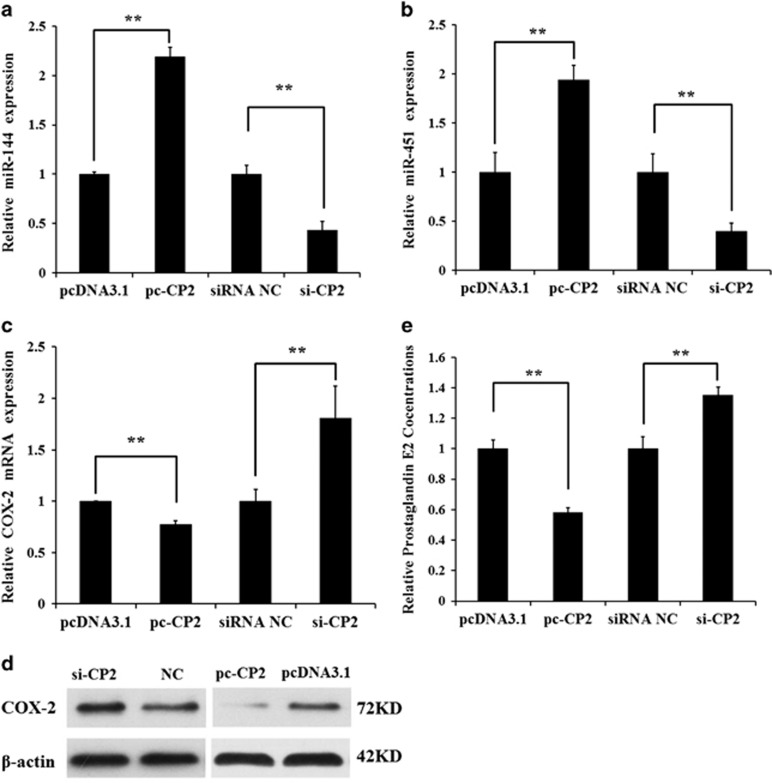
CP2 promotes the expression of miR-144 and *COX-2*. (**a**) qRT-PCR was used to detect endogenous miR-144 level 48 h after mGCs were transfected with *pcDNA3.1-CP2*, *pcDNA3.1*, siRNA-CP2 or siRNA NC. (**b**) qRT-PCR was used to detect endogenous miR-451 levels 48 h after mGCs were transfected with *pcDNA3.1-CP2*, *pcDNA3.1*, siRNA-CP2 or siRNA NC. (**c**) qRT-PCR was used to detect endogenous *COX-2* mRNA levels 48 h after mGCs were transfected with *pcDNA3.1-CP2*, *pcDNA3.1*, siRNA-CP2 or siRNA NC. (**d**) Western blot analysis was used to detect endogenous COX-2 protein levels 72 h after mGCs were transfected with *pcDNA3.1-CP2*, *pcDNA3.1*, siRNA-CP2 or siRNA NC. (**e**) The PGE2 levels in the culture media were examined by ELISA. Mouse GCs were transfected with *pcDNA3.1-CP2*, *pcDNA3.1*, siRNA-CP2 or siRNA NC. After 48 h, cell media were collected to measure PGE2 levels using an ELISA kit. The results are expressed as the mean±S.E.M. (three independent replicates per group). **P<0.01

To further verify that CP2 regulates miR-144 expression, the *pcDNA3.1-CP2* vector or siRNA-CP2 was transfected into mGCs, respectively. CP2 overexpression significantly promoted miR-144 as determined by qRT-PCR analysis, whereas knockdown of CP2 suppressed miR-144 expression (Figure 6a). There is increasing evidence that miR-144 and miR-451 are transcribed on a single primary RNA and regulated by same transcription factors.^23, 24, 25^ We hypothesize that CP2 can also regulate miR-451 expression in mGCs. As shown in Figure 6b, CP2 significantly promoted miR-451 expression.

As *COX-2* was identified as a direct target of miR-144, and the transcription factor CP2 upregulated mature miR-144 expression, we hypothesize that CP2 can affect *COX-2* expression and PGE2 production. To test this hypothesis, *pcDNA3.1-CP2*, *pcDNA3.1*, siRNA-CP2 or siRNA NC was transfected into mGCs. As shown in Figures 6c and e, CP2 markedly suppressed endogenous *COX-2* expression levels and PGE2 production. These results indicate that CP2 binds to the core promoter of miR-144, induces the expression of mature miR-144 and miR-451, and eventually suppresses *COX-2* expression and PGE2 production.

### miR-144 regulates mGC apoptosis but not via *COX-2* and *Smad4*

**Figure 7:**
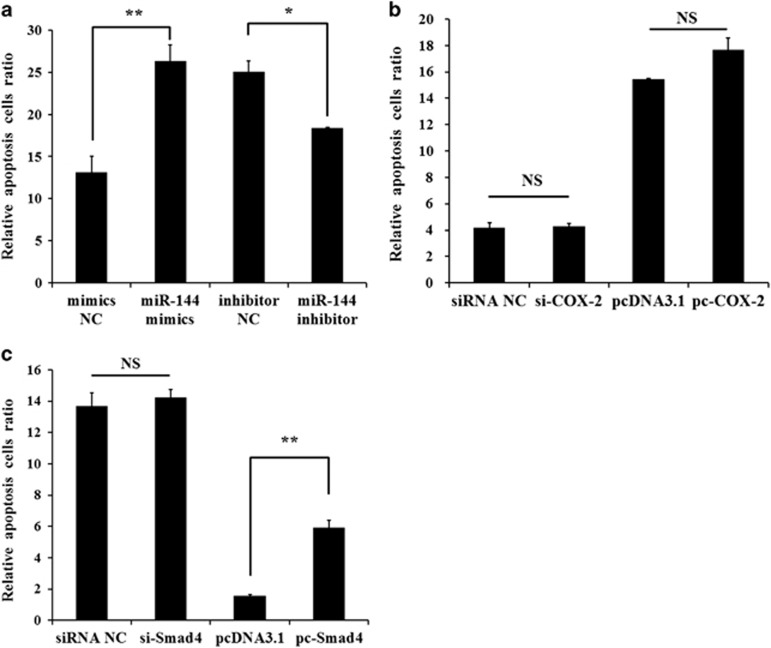
miR-144 regulated mGC apoptosis. (**a**) Mouse GCs were transfected with miR-144 mimics, mimics NC, miR-144 inhibitor, or inhibitor NC, harvested and stained with anti-annexin V-propidium iodide, and analyzed by FACS after 48 h. (**b**) Mouse GCs were transfected with siRNA-COX-2, siRNA NC, *pcDNA3.1-COX-2* or *pcDNA3.1*, harvested and stained with anti-annexin V-propidium iodide, and then analyzed by FACS after 48 h. (**c**) Mouse GCs were transfected with siRNA-Smad4, siRNA NC, *pcDNA3.1-Smad4* or *pcDNA3.1*, harvested and stained with anti-annexin V-propidium iodide, and then analyzed by FACS after 48 h. The results are expressed as the mean±S.E.M. (three independent replicates per group). *P<0.05, **P<0.01, N.S.=nonsignificant

Previous studies indicate that follicular atresia is triggered by granulosa cell apoptosis.^18, 26, 27^ We hypothesize that miR-144 can affect apoptosis of mGCs. miR-144 mimics, mimics NC, miR-144 inhibitor or inhibitor NC was transfected into mGCs. As shown in Figure 7a, Supplementary Figures S7 and S8, miR-144 overexpression promoted apoptosis of mGCs, and miR-144 inhibition suppressed mGC apoptosis. Our studies demonstrated that *Smad4* and *COX-2* were two target genes of miR-144. Thus, miR-144 may regulate mGC apoptosis through *Smad4* or *COX-2*. However, either overexpression or inhibition of *COX-2* had no effect on apoptosis of mGCs (Figure 7b,Supplementary Figures S9 and S10). Although *Smad4* overexpression promoted mGC apoptosis, *Smad4* inhibition had no effect on mGC apoptosis (Figure 7c, Supplementary Figures S11 and S12). These results indicate that miR-144 regulates mGC apoptosis but not through *Smad4* and *COX-2*.

## Discussion

Mammalian folliculogenesis is a complex biological process. Initially primordial follicles develop into pre-ovulatory follicles, and then ovulation occurs. Ovulation is regulated by hormones and other external cues.^28^ However, the regulatory mechanism underlying ovulation remains unclear.

Many previous studies have indicated that miRNAs have a vital role in the folliculogenesis. Deletion of Dicer in mGCs lead to female sterility and multiple reproductive defects.^29^ miR-200b and miR-429 knockout mice have lower serum LH concentrations, an impaired LH surge, and failure to ovulate.^30^ Androgens attenuates follicular atresia by enhancing expression of miR-125b, which suppresses pro-apoptotic protein expression.^17^ miR-224 regulates mouse cumulus expansion by targeting pentraxin 3 (*PTX3*) *in vivo* and *in vitro*.^31^ In this study, we used Solexa deep sequencing and bioinformatics analysis to define 390 known miRNAs differentially expressed between pre-ovulatory ovarian follicles of CT and LW sows. The expression patterns of seven known miRNAs (let-7a, miR-125a, miR-144, miR-2423, miR-3613-5p, miR-331* and miR-4028-3p) were successfully validated using qRT-PCR analyses. miRNA microarrays show that let-7 family members are differentially expressed during follicular atresia, and further research demonstrate that let-7 g induces porcine granulosa cell apoptosis by targeting mitogen-activated protein kinase kinase kinase 1 (*MAP3K1*) in the porcine ovary.^18, 32^ miR-125a-5p is shown to regulate mGC apoptosis by targeting signal transducer and activator of transcription 3 (*STAT3*), and miR-125a-3p reduces the number of ovulated oocytes by targeting the Fyn proto-oncogene (*Fyn*).^33, 34^ However, many studies on miR-144 focus on cancer cell proliferation, invasion and erythropoiesis, and few studies have examined the role of miR-144 in folliculogenesis.^35, 36, 37, 38^

Importantly, we revealed that the *COX-2* gene was a target of miR-144 in both mGCs and PK-15 cells. *COX-2* is primarily produced from the secondary follicle stage to the Graafian follicle stage. The production of this enzyme is stopped after the Graafian follicle stage and is resumed with exposure to the LH surge.^39^ *COX-2*-deficient mice have poor ovulation and low fertilization rates.^14^ Moreover, ovulation can be restored by treatment of the *COX-2-*deficient mice with PGE2.^15^ *COX-2*-derived PGE2 is essential for ovulation because it activates PGE2, which is involved in the MAPK, NF-*κ*B and phosphatidylinositol 3-kinase/Akt pathways.^40, 41^ PGE2 upregulates EGF-like growth factor biosynthesis, and EGF-like growth factors induce *COX-2* expression and PGE2 production by activating the ERK1/2 signalling pathway in human granulosa cells.^42, 43^ We confirm that miR-144 suppresses PGE2 production by targeting *COX-2*. Therefore, this study provides a new mechanism by which miR-144 regulates ovulation via targeting *COX-2* and then suppressing PGE2 production.

Many studies have shown that the TGF-*β* signalling pathway regulates ovulation. TGF-*β*1 induces *COX-2* expression and PGE2 production by activating Smad signalling pathways in human granulosa cells.^22^ TGF-*β*1 induces mGC proliferation by upregulating miR-224 expression.^44^ TGF-*β*1 downregulates steroidogenic acute regulatory protein (*StAR*) expression and decreases progesterone production by activating the Smad3 and ERK1/2 signalling pathways in human granulosa cells.^45^ In our study, we demonstrate that *Smad4* is a target of miR-144 in mGCs, and *Smad4* regulates *COX-2* expression via the TGF-*β* signalling pathway. A previous study suggests that miR-144 regulates *COX-2* by targeting *c-Fos*.^46^ We found that miR-144 regulated *c-Fos* expression in mGCs directly through dual-luciferase reporter assays, qRT-PCR and western blot analyses (Supplementary Figure S13). However, *c-Fos* had no significant effect on *COX-2* expression in mGCs (Supplementary Figure S14). The contrasting results between human amniotic epithelial cells (WISH) and mGCs are probably due to the different sources of these two cells. These studies further confirm that miR-144 regulates *COX-2* expression indirectly by targeting *Smad4*, but not *c-Fos*, in mGCs.

Several previous studies have indicated that miRNA expression is also regulated by transcription factors in granulosa cells.^47, 48^ Steroidogenic factor-1 suppresses miR-383 transcription and then mediates oestradiol release in mGCs.^47^ TGF-*β*1 enhances the binding of p53 and NF-κB p65 to the promoter region of the miR-244 host gene, promotes miR-244 host gene and pri-miR-244 transcription, and affects mGC proliferation and oestradiol release in mGCs.^48^ In this study, we find that transcription of miR-144 is regulated by CP2 in both mGCs and CHO-K1 cells. Previous studies of CP2 focus on *α*-globin transcriptional regulation and Alzheimer's disease, while few studies have investigated its role in reproduction.^49, 50, 51, 52^ A mutated CP2-binding site in the LH-*β* promoter region may result in a higher litter size of D'man sheep than Sardi sheep and Timahdite sheep.^53^ CP2 can be used as a potential diagnostic biomarker of ovarian cancer.^54^ CP2 is an essential transcription factor for the regulation of sex determining region Y (*SRY*) expression, and *SRY* can affect sex determination in mammals.^55^ We further demonstrate that CP2 affects PGE2 production through the CP2/miR-144/*COX-2*/PGE2 axis. These results suggest that CP2 has an important role in ovulation.

miR-144 and miR-451 are transcribed in the same pri-miRNA.^25^ Paired box gene 4 (PAX4), which regulates human epithelial cancer metastasis, decreases miR-144 and miR-451 expression levels by binding to the promoter region of miR-144/451.^25^ GATA-binding protein 4 (GATA4) has been shown to activate the promoter of miR-144/451 and protect against simulated ischaemia/reperfusion-induced cardiomyocyte death.^24^ Here, we find that CP2 regulates the expression of miR-144 and miR-451 in mGCs, which is consistent with previous studies.

Many factors, such as tumour necrosis factor (*TNF*) and X-linked inhibitor of apoptosis protein (*XIAP*), are involved in follicular atresia in pigs.^56, 57^ miR-26b promotes porcine granulosa cell apoptosis and induces follicular atresia by targeting ataxia telangiectasia mutated (*ATM*) in porcine ovary.^58^ let-7 g targets *MAP3K1* and TGF-*β* type 1 receptor (*TGFBR1*) and regulates porcine granulosa cell apoptosis.^18, 26^ In this study, we demonstrate that miR-144 regulates apoptosis of mGCs but not via *Smad4* and *COX-2*. These data indicate that miR-144 may be involved in follicular atresia.

**Figure 8:**
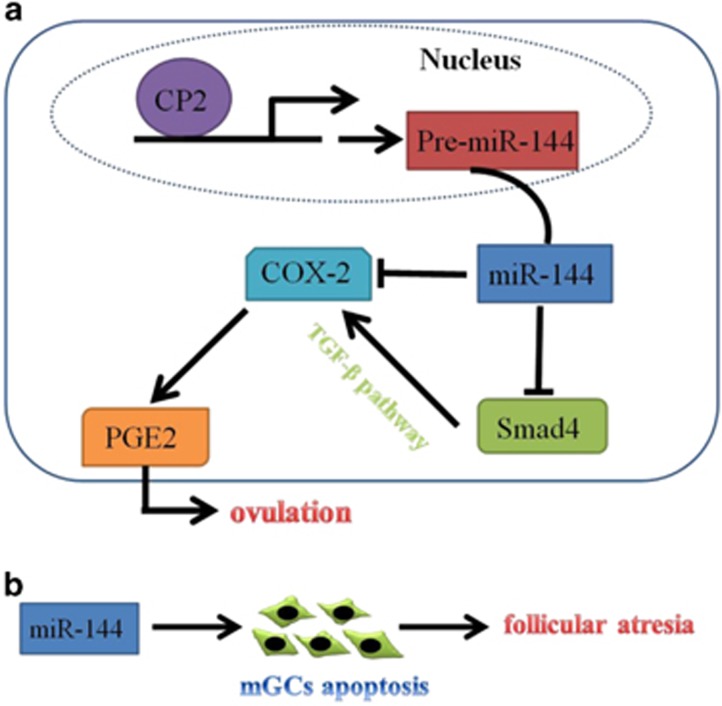
A graphic abstract showing the major findings of this study. (**a**) Schematic diagram of the CP2/miR-144/*COX-2*/PGE2 pathway in mGCs and its roles in ovulation. (**b**) MiR-144 regulated mGC apoptosis and then affected follicular atresia

In summary, our study provides direct evidence that miR-144 participates in mammalian ovulation by regulating PGE2 production. The potential mechanism underlying the suppression of PGE2 production by miR-144 involves direct targeting of *COX-2* and *Smad4* genes. In addition, miR-144 was upregulated by CP2 (Figure 8a). We also showed that miR-144 regulated mGC apoptosis but not through *Smad4* or *COX-2* (Figure 8b). These results suggest that a novel signalling pathway involves in ovulation and follicular atresia. These findings have potential implications in improving female fecundity through the CP2/miR-144/*COX-2*/PGE2/ovulation pathway.

## Materials and Methods

### Animals and tissues

Three multiparous CT cyclic sows (≥2 parities) from Xuqin Corporation (Changshu, Jiangsu, China) and three multiparous LW cyclic sows (≥2 parities) from the Jinpin farm of Huazhong Agricultural University, Wuhan, China that exhibited normal oestrous cycles were treated as reported previously.^59^ Briefly, the sows were injected with 1000 IU pregnant mare serum gonadotropin (PMSG) (SanSheng, Ningbo, Zhejiang, China) and 500 IU hCG (SanSheng) as described previously.^60^ Only healthy ovarian follicles with a diameter of >5 mm were isolated and snap-frozen in liquid nitrogen. Total RNA was isolated from ovarian follicles with TRIzol reagent (Invitrogen, Carlsbad, CA, USA). Twenty- to 22-day-old Kunming White female mice used in this study were obtained from the Centre of Laboratory Animals of Hubei Province (Wuhan, Hubei, China). All animal treatment procedures were approved by the ethical committee of the Hubei Research Centre of Experimental Animals (approval ID: SCXK (Hubei) 2008-0005).

### Solexa sequencing

A RNA pool from LW pre-ovulatory ovarian follicles and a RNA pool from Taihu pre-ovulatory ovarian follicles were submitted to Illumina sequencing as described previously.^61^ Solexa sequencing was carried out at the Beijing Genomics Institute (BGI). Raw data from the Illumina 1G Genome Analyser were processed using Solexa software (Illumina). Low-quality reads were filtered according to the base quality value.

After redundancies were removed, sequences ≥18 nt were perfectly mapped to the swine genome Sscrofa10.2 using SOAP. The unique sequences were compared with the miRNA database, miRBase 16.0 (http://www.mirbase.org/), by BLASTn search to identify the conserved miRNAs in pigs. The differentially expressed small non-coding RNAs were identified by comparing their expression between a RNA pool from LW pre-ovulatory ovarian follicles and a RNA pool from Taihu pre-ovulatory ovarian follicles.^61^

### Cell culture, transfection and dual-luciferase reporter assays

Granulosa cells from pre-ovulatory ovarian follicles were obtained from murine ovaries after Kunming White female mice were treated for 48 h with 10 IU PMSG (SanSheng). The CHO-K1 cells (GDC018) and PK-15 cells (GDC061) were obtained from the China Centre for Type Culture Collection (Shanghai, China). Cells were cultured with Dulbecco's minimum essential medium/nutrient F-12 (DMEM/F-12) (Gibco, Waltham, MA, USA) or DMEM (Gibco) supplied with 10% foetal bovine serum (Gibco) at 37 °C in a humidified atmosphere of 5% CO_2_. The cells were plated and grown until they were 70–80% confluent at the time of transfection. miRNA or siRNA was transfected into the cells using Lipofectamine RNAiMAX (Invitrogen). Plasmids were transfected into the cells using Lipofectamine 3000 (Invitrogen). For luciferase assays, each wild-type or deletion construct was transfected into the cells at 500 ng, together with 10 ng/well of pRL-TK (Promega, Madison, WI, USA). After transfection for 24 h, the luciferase activities were measured with a PerkinElmer 2030 Multilabel Reader (PerkinElmer, Waltham, MA, USA).

### Plasmid constructs

Nine deletion fragments of the mouse miR-144 potential promoter region were amplified and double-digested with *Kpn*I and *Hind*III and cloned into the pGL3-basic vector (Promega). Mouse *COX-2* (NM_011198.4), *Smad4* (NM_008540.2) and *c-Fos* (NM_010234.2) CDS were amplified and double-digested with *Nhe*I and *Hind*III and cloned into the *pcDNA3.1(+)* vector (Invitrogen). The mouse *CP2* (NM_001289603.1) CDS was amplified, double-digested with *Hind*III and *Xba*I, and then cloned into the pCMV-C-HA vector (Beyotime, Shanghai, China). The mouse *COX-2*, *Smad4* and *c-Fos* 3′-UTRs were amplified, double-digested with *Pme*I and *Xho*I, and then cloned into the pmirGLO vector (Promega). Binding site mutants were generated using a MutanBEST Kit (TaKaRa, Dalian, Liaoning, China) and mutagenic primers. The primers are described in Supplementary Table S2.

### RNA interference

The synthesized siRNA-CP2, siRNA-COX-2, siRNA-Smad4 siRNA-c-Fos or siRNA NC (RiboBio, Guangzhou, Guangdong, China) was transfected into cells with Lipofectamine RNAiMAX (Invitrogen). After 48 and 72 h, cells were harvested for qRT-PCR assays and western blot assays, respectively. The siRNA sequences are as follows: siRNA-CP2 sense sequence: 5′-CCAUAGCAGUUUCUCUCUUdTdT-3′ antisense sequence: 3′-dTdTGGUAUCGUCAAAGAGAGAA-5′ the siRNA-COX-2 sense sequence: 5′-CACAGGAUUUGACCAGUAUdTdT-3′ antisense sequence: 3′-dTdTGUGUCCUAAACUGGUCAUA-5′ the siRNA-Smad4 sense sequence: 5′-ACAGUGAAGGUGAAUUAAAdTdT-3′ antisense sequence: 3′-dTdTUGUCACUUCCACUUAAUUU-5′ siRNA-c-Fos sense sequence: 5′-GGAGACAGAUCAACUUGAAdTdT-3′ antisense sequence: 3′-dTdTCCUCUGUCUAGUUGAACUU-5′.

### qRT-PCR analysis

Total RNA (including miRNA) was extracted from tissues or cells with TRIzol reagent (Invitrogen). Cellular RNA was extracted 48 h after transfection. Primers used in the qRT-PCR are shown in Supplementary Table S2. One microgram of RNA was treated with 1 *μ*l DNase I (Fermentas, St. Leon-Rot, Germany) to remove DNA contamination. Reverse transcription was performed using a RevertAid First Strand cDNA Synthesis Kit (Thermo Fisher Scientific, Waltham, MA, USA). Random primers, oligo(dT)_18_ or miRNA specific stem-loop primers were added to initiate cDNA synthesis. qRT-PCR was performed on a Bio-Rad CFX96 system (Bio-Rad, Richmond, CA, USA) using the iTaq Universal SYBR Green Supermix (Bio-Rad). All PCR reactions were performed in triplicate. Gene expression levels were normalized to the expression of *β*-actin, and miRNA expression levels were normalized to the expression of U6 using Gene Expression Macro software (Bio-Rad) by using the 2^−ΔΔCt^ method.^61^

### Western blot analysis

Cell protein lysates were generated using RIPA Lysis Buffer (Beyotime). Cellular proteins were extracted 72 h after transfection. Proteins were separated by SDS-PAGE, and a Mini Trans-Blot Cell (Bio-Rad) was used to transfer protein onto polyvinylidene fluoride membranes (Millipore, Billerica, MA, USA). Primary antibodies specific for COX-2 (1:1000; Santa Cruz Biotechnology, St. Louis, MO, USA, sc-1746), Smad4 (1:2000; Abcam, Cambridge, MA, USA, ab40759), c-Fos (1:3000; Abcam, ab134122), *β*-actin (1:2000; Santa Cruz Biotechnology, ab8226) and CP2 (1:500; Proteintech, Wuhan, Hubei, China, 15203-1-AP) were used for immunoblotting. An Image Quant LAS4000 mini (GE Healthcare Life Sciences, Piscataway, NJ, USA) was used to detect protein expression.

### Sequence analysis

The BIOBASE software (http://www.gene-regulation.com/pub/ programs.html) was used to predict the transcription factor binding sites in the promoters of both mouse miR-144 and pig miR-144. Neural network promoter prediction software (http://www.fruitfly.org/seqtools/promoter.html) was used to analyze the potential promoters, and TargetScan (http://www.targetscan.org/) was used to predict the potential target genes of miRNAs.

### Chromatin immunoprecipitation

As specifical immunoprecipitate CP2 antibodies are not available, we constructed *pCMV-C-HA*-CP2-CDS and then transfected this vector into mGCs. ChIP was performed using the EZ-ChIP Kit (Millipore). The AVCX130 system (Sonics & Materials, Newtown, CT, USA) was used for cell sonication. Anti-HA (Abcam, ab9110), anti-OCT1 (Santa Cruz Biotechnology, sc-25399 X) and normal anti-mouse-IgG (Millipore) were used for the immunoprecipitation reactions. DNA from the immunoprecipitated complex was amplified via PCR. The primer sequences are described in Supplementary Table S2.

### Electrophoretic mobility shift assay

EMSA was performed as described previously.^62^ Briefly, *pCMV-C-HA-CP2-CDS* was transfected into mGCs, and then, the nuclear extracts were isolated with a Nucleoprotein Extraction Kit (Beyotime). Oligos corresponding to the CP2-binding sites of the miR-144 core promoter were synthesized and annealed into double strands. The DNA-binding activity of CP2 protein was detected by LightShift Chemiluminescent EMSA Kit (Thermo Fisher Scientific).

### Enzyme-linked immunosorbent assay (ELISA)

Mouse GCs were cultured in a six-well plate with 2 ml medium. Mouse GCs were transfected with oligonucleotides or plasmids. After 48 h, cell media were collected to measure PGE2 levels using an ELISA kit (Enzo Life Sciences, Farmingdale, NY, USA). PGE2 levels were normalized to the protein concentrations. Normalized PGE2 values were determined by extrapolating from a standard curve.

### Cell apoptosis analysis

Mouse GCs were transfected with miR-144 mimics, mimics NC, miR-144 inhibitor or inhibitor NC and harvested 48 h after transfection. Fluorescence-activated cell sorting (FACS) was used to measure apoptosis. The experiments were performed according to the manufacturer's protocol of the Annexin V-FITC Apoptosis Detection Kit (Invitrogen).

### Statistical analysis

All results are presented as the mean±S.D. Each treatment had three replicates. Two-tailed *t*-test was used when two groups were compared. Significant differences were evaluated using an independent-samples *t*-test. *P*<0.05 was considered to be statistically significant.
