# Supplementary material for: MicroRNA-144 is regulated by CP2 and decreases COX-2 expression and PGE2 production in mouse ovarian granulosa cells
Source: Cell Death Dis. 2017 Feb 9;8(2):e2597–. doi: 10.1038/cddis.2017.24 (PMC5386473; doi:10.1038/cddis.2017.24)
Supplement: Supplementary Figures [file cddis201724x1.doc]

**
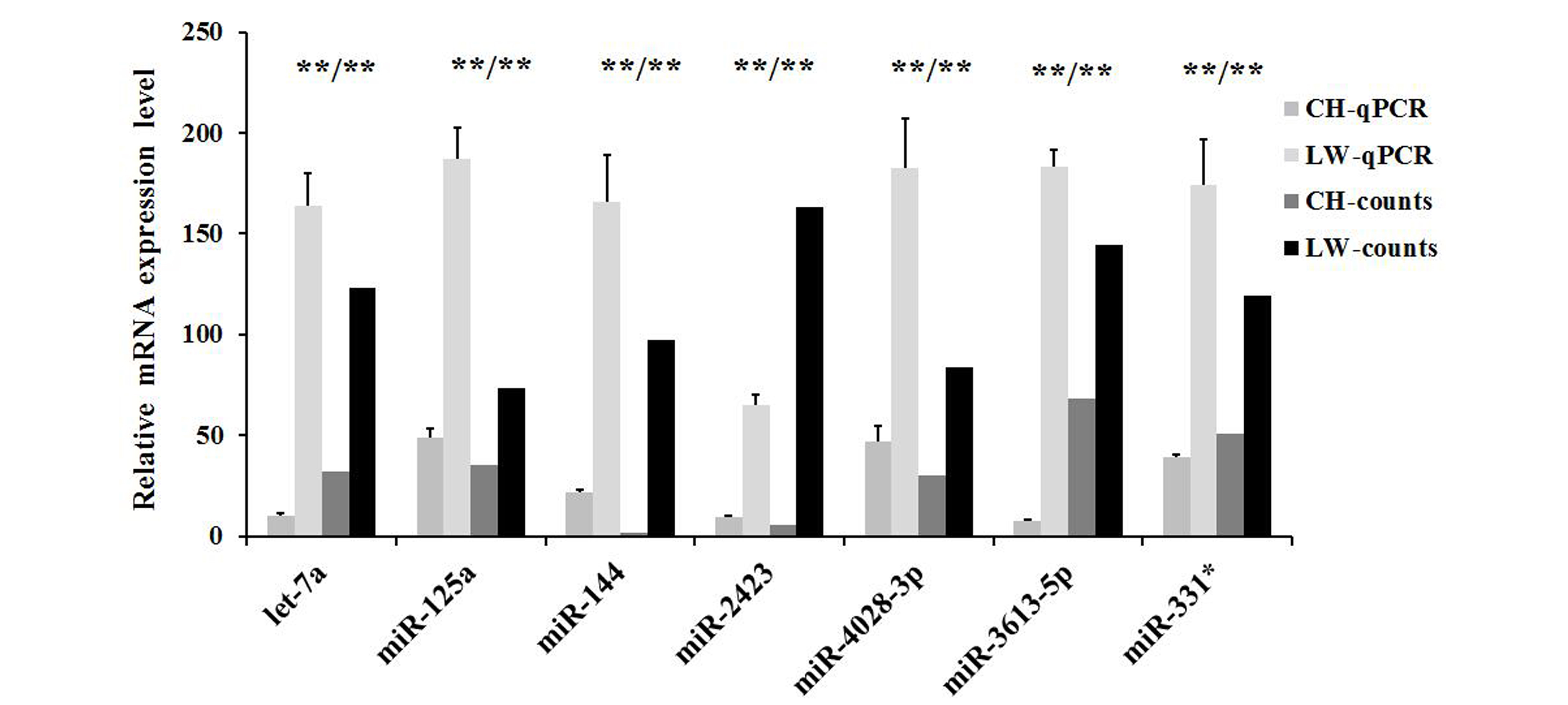
**

**Figure S1** Quantitative RT-PCR validation of the differential expression of miRNAs in the pre-ovulatory ovarian follicles of Chinese Taihu and Large White sows. The expression levels of let-7a, miR-125a, miR-144, miR-2423, miR-4028-3p, miR-3613-5p and miR-331* were detected by qRT-PCR in the pre-ovulatory ovarian follicles of Chinese Taihu and Large White sows. The results are expressed as the mean ± S.E.M. (three independent replicates per group). ***P*<0.01


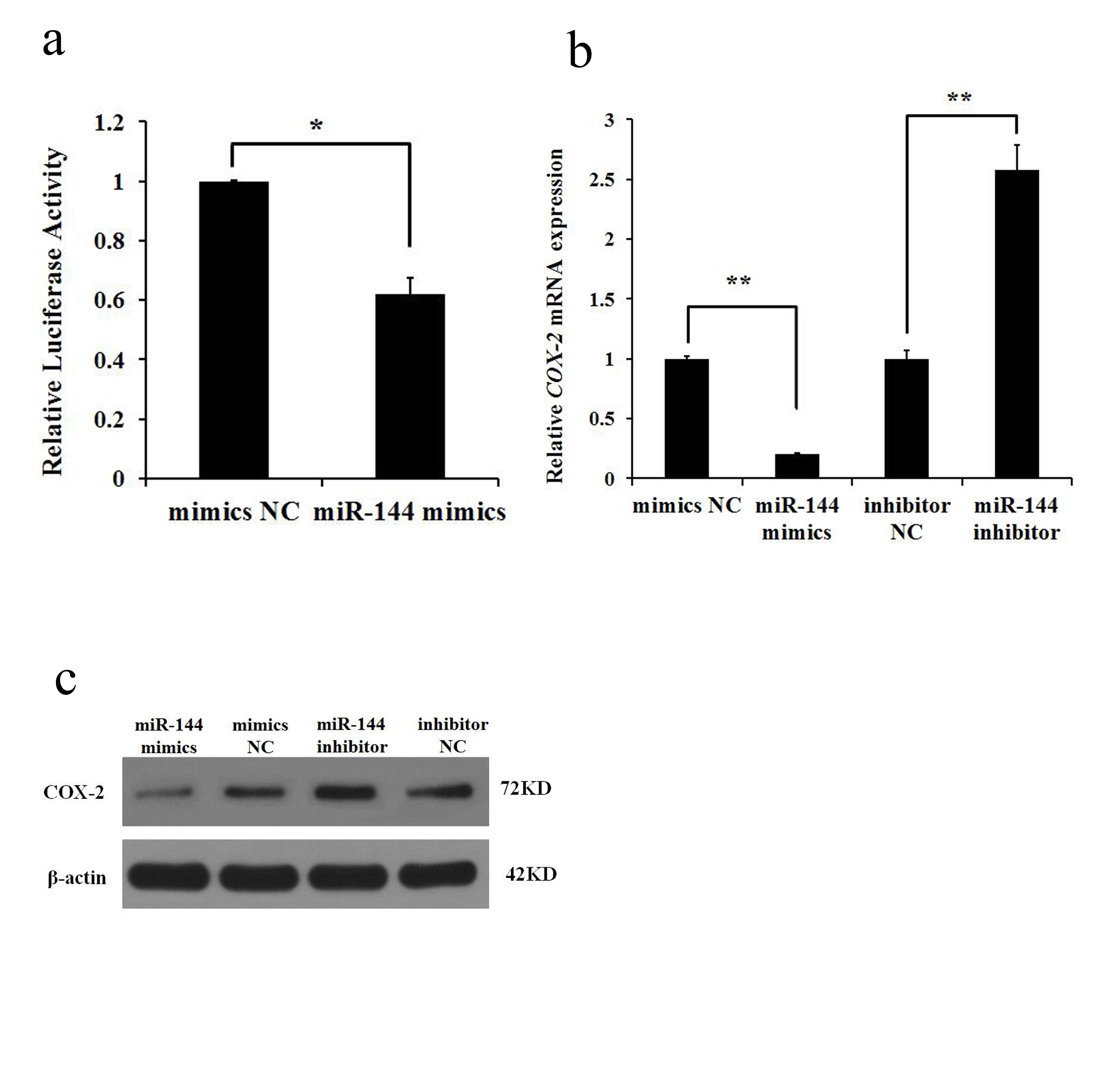


**Figure S2** *COX-2* as a direct target of miR-144 in PK-15 cells. (**a**) Luciferase activity was analyzed after cells were co-transfected with *pmiRGLO-COX-2-3’UTR* and miR-144 mimics or mimics NC. (**b**) Endogenous *COX-2* mRNA level was detected after mGCs were transfected with miR-144 mimics, mimics NC, miR-144 inhibitor or inhibitor NC. (**c**) Western blot was used to detect endogenous COX-2 protein expression level after mGCs were transfected with miR-144 mimics, mimics NC, miR-144 inhibitor or inhibitor NC. The results are expressed as the mean ± S.E.M. (three independent replicates per group). **P*<0.05, ***P*<0.01


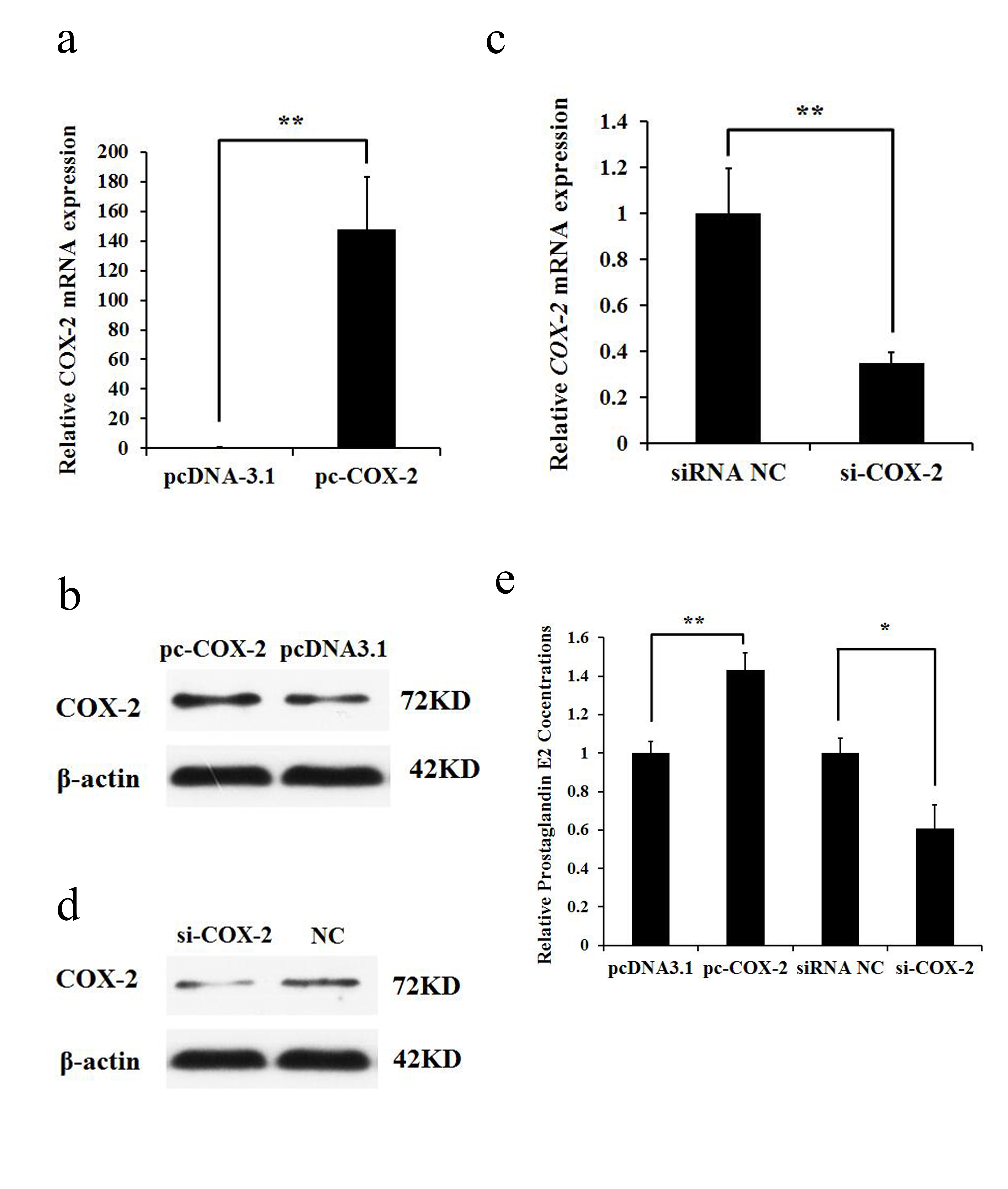


**Figure S3** *pcDNA3.1-COX-2* and siRNA-COX-2 could increase or reduce *COX-2* expression levels in mGCs, respectively. (**a**) The *pcDNA3.1-C0X-2* or *pcDNA3.1* was transfected into mGCs, and *COX-2* mRNA expression level was measured by qRT-PCR. (**b**) The *pcDNA3.1-C0X-2* or *pcDNA3.1* was transfected into mGCs, and COX-2 protein expression level was measured by Western blot. (**c**) The siRNA-COX-2 or siRNA NC was transfected into mGCs, and *COX-2* mRNA expression level was measured by qRT-PCR. (**d**) The siRNA-COX-2 or siRNA NC was transfected into mGCs, and COX-2 protein expression level was measured by Western blot. (**e**) Mouse GCs were transfected with *pcDNA3.1-C0X-2*, *pcDNA3.1*, siRNA-COX-2 or siRNA NC, and measured by ELISA kit after 48 h. The results are expressed as the mean ± S.E.M. (three independent replicates per group). **P*<0.05, ***P*<0.01


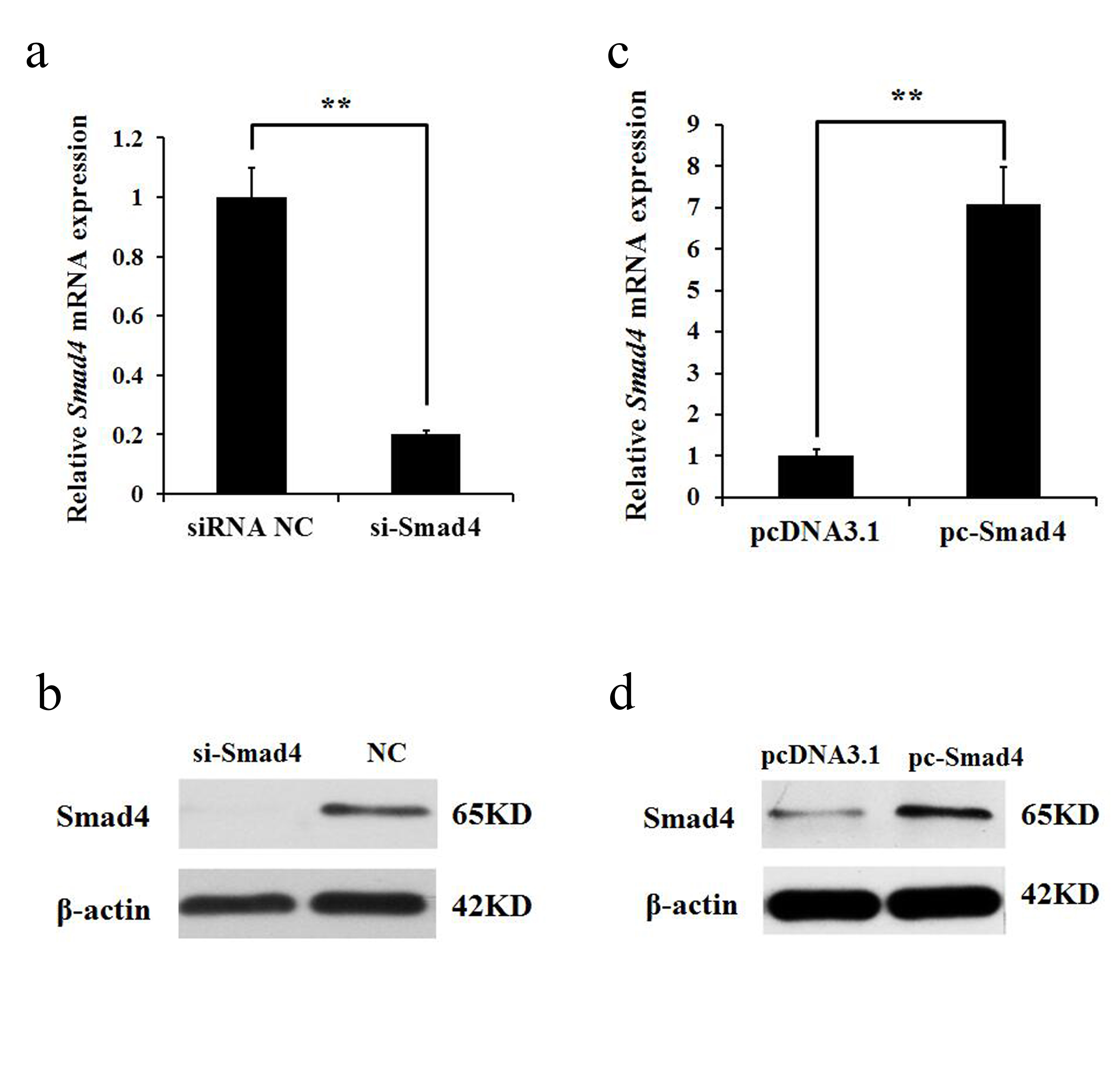


**Figure S4** *pcDNA3.1-Smad4* and siRNA-Smad4 could increase or reduce *Smad4* expression levels in mGCs, respectively. (**a**) The siRNA-Smad4 or siRNA NC was transfected into mGCs, and *Smad4* mRNA expression level was measured by qRT-PCR. (**b**) The siRNA-Smad4 or siRNA NC was transfected into mGCs, and Smad4 protein expression level was measured by Western blot. (**c**) The *pcDNA3.1-Smad4* or *pcDNA3.1* was transfected into mGCs, and *Smad4* mRNA expression level was measured by qRT-PCR. (**d**) The *pcDNA3.1-Smad4* or *pcDNA3.1* was transfected into mGCs, and Smad4 protein expression level was measured by Western blot. The results are expressed as the mean ± S.E.M. (three independent replicates per group). ***P*<0.01


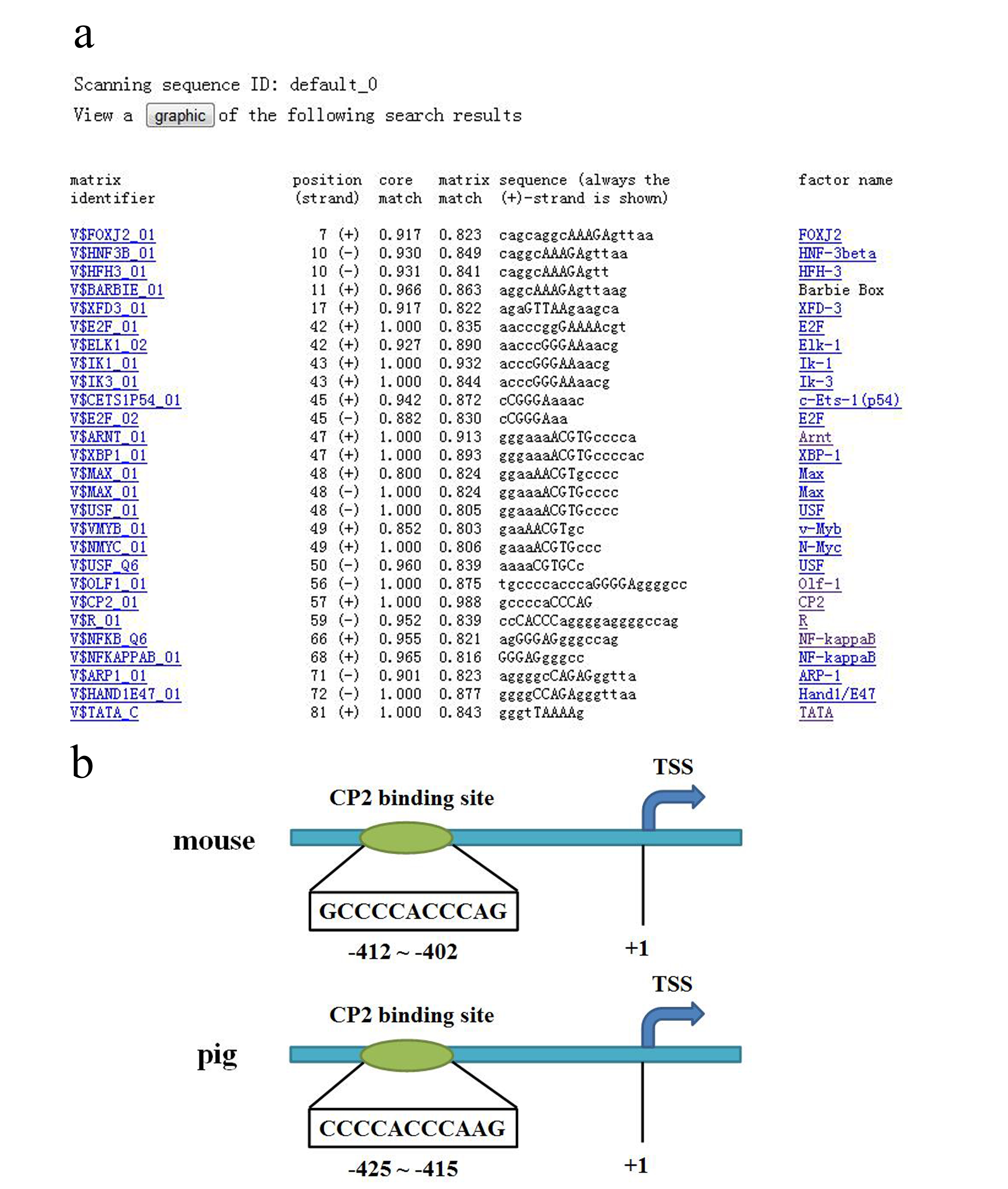


**Figure S5** BIOBASE and NCBI software were used to predict CP2 might bind to the miR-144 promoter in mouse and pig. (**a**) BIOBASE software to used determine the potential transcription factor binding sites with the core match and matrix match at least 0.8. (**b**) Sequence analysis of CP2 binding sites of miR-144 promoter between mouse and pig


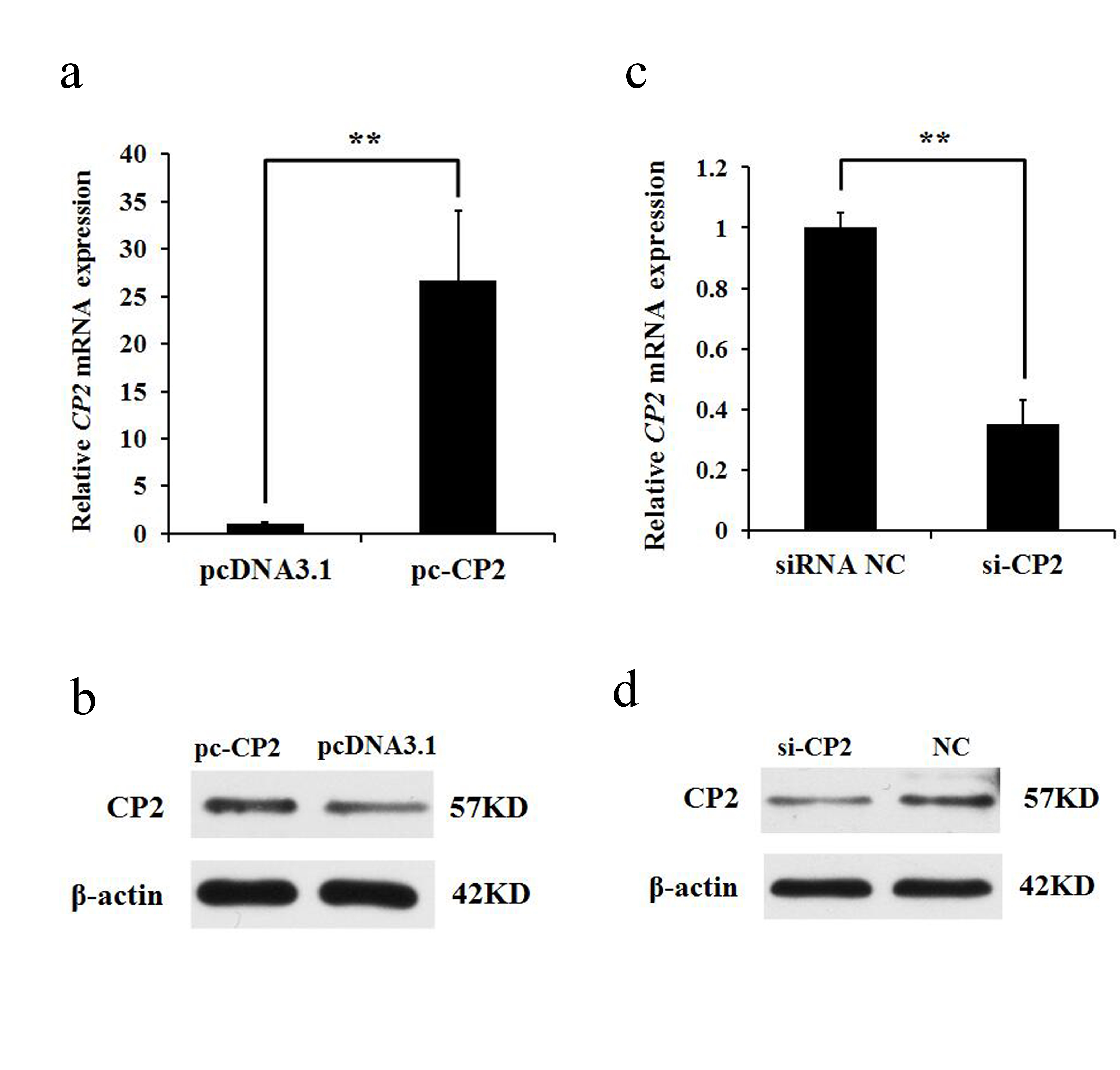


**Figure S6** *pcDNA3.1-CP2* and siRNA-CP2 could increase or reduce CP2 expression levels in mGCs, respectively. (**a**) The *pcDNA3.1-CP2* or *pcDNA3.1* was transfected into mGCs, and CP2 mRNA expression level was measured by qRT-PCR. (**b**) The *pcDNA3.1-CP2* or *pcDNA3.1* was transfected into mGCs, and CP2 protein expression level was measured by Western blot. (**c**) The siRNA-CP2 or siRNA NC was transfected into mGCs, and *CP2* mRNA expression level was measured by qRT-PCR. (**d**) The siRNA-CP2 or siRNA NC was transfected into mGCs, and CP2 protein expression level were measured by Western blot. The results are expressed as the mean ± S.E.M. (three independent replicates per group). ***P*<0.01


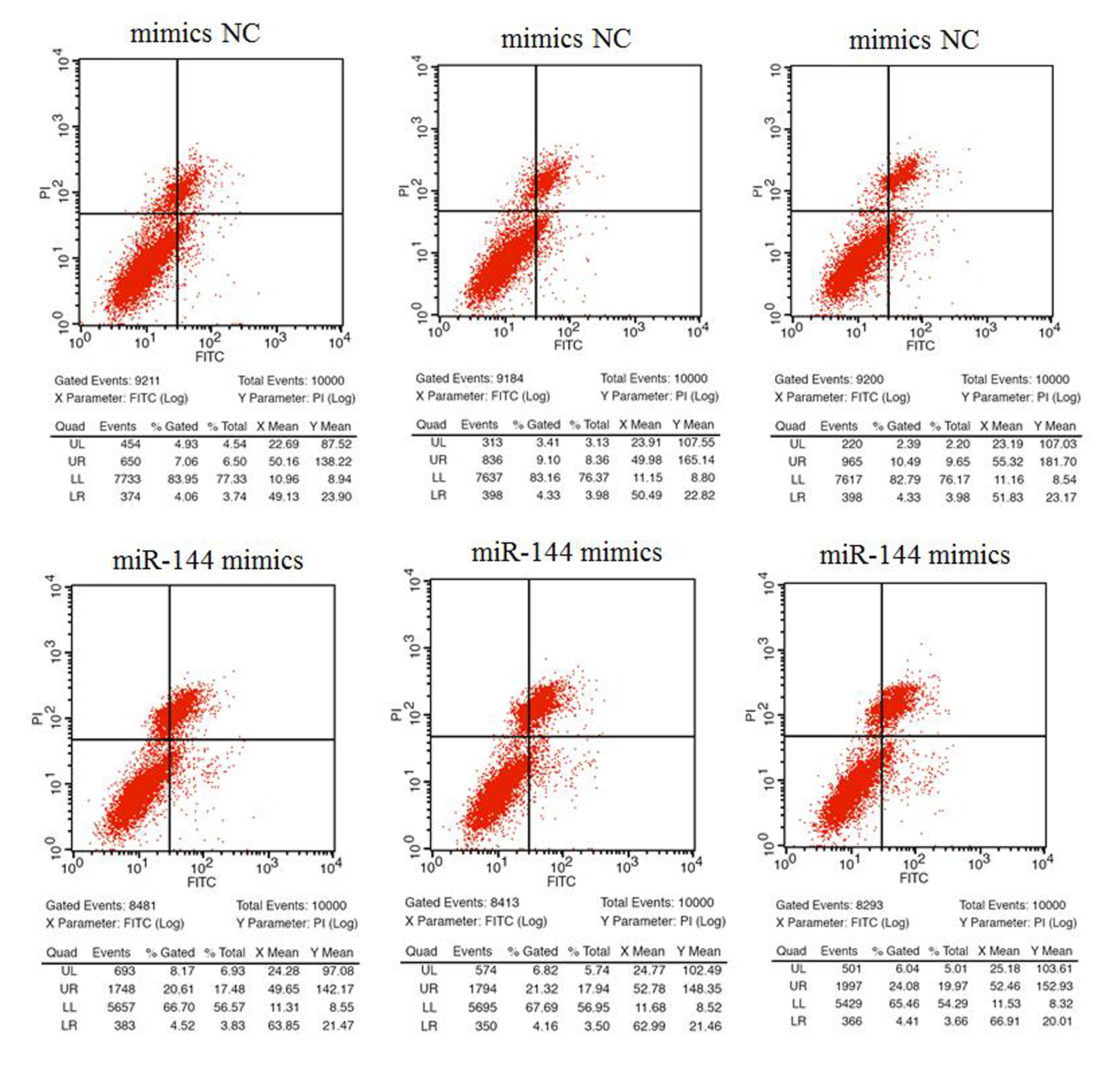


**Figure S7** Flow cytometry was used to detect mGC apoptosis after mGCs were transfected with miR-144 mimics or mimics NC


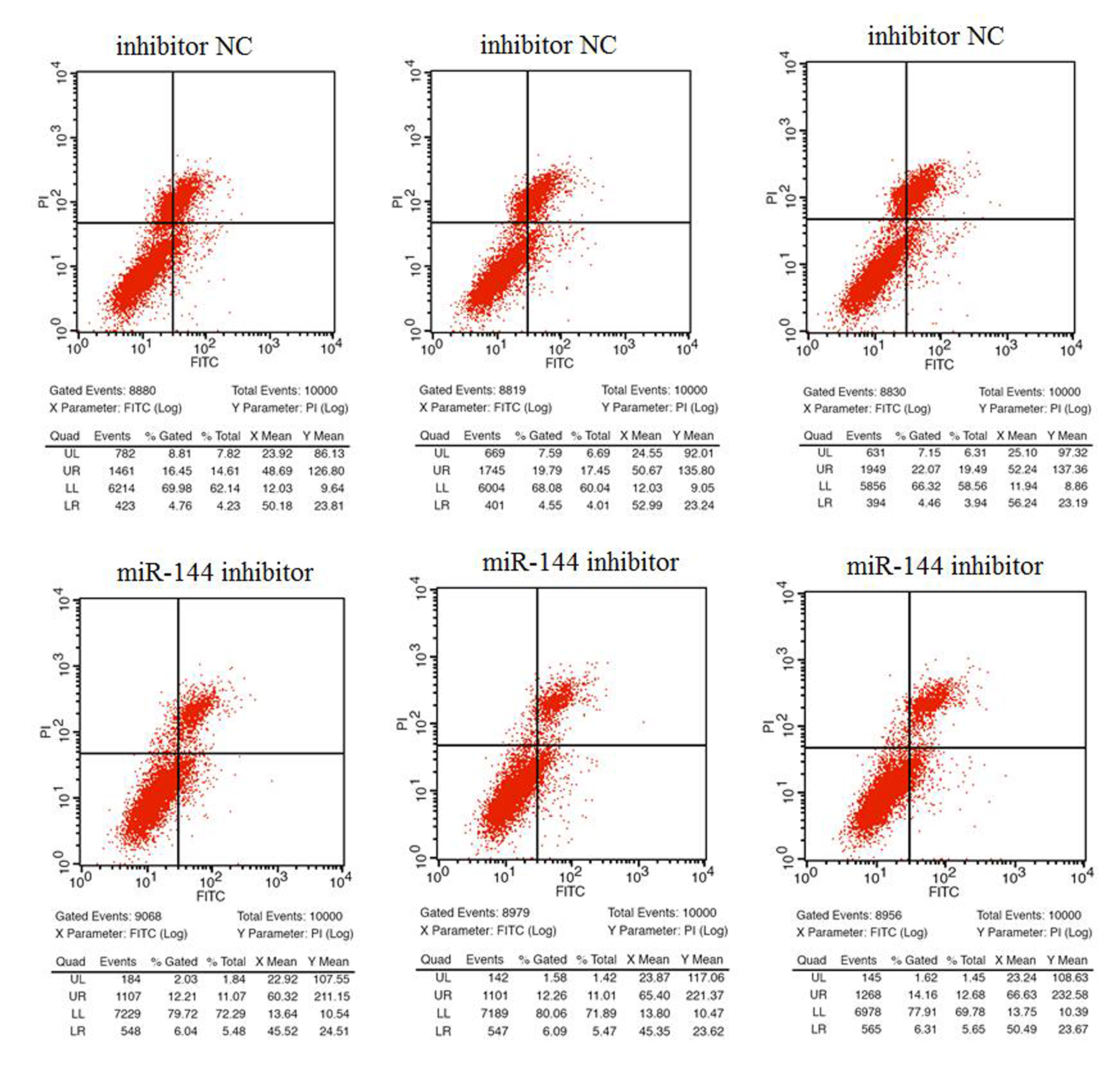


**Figure S8** Flow cytometry was used to detect mGC apoptosis after mGCs were transfected with miR-144 inhibitor or inhibitor NC


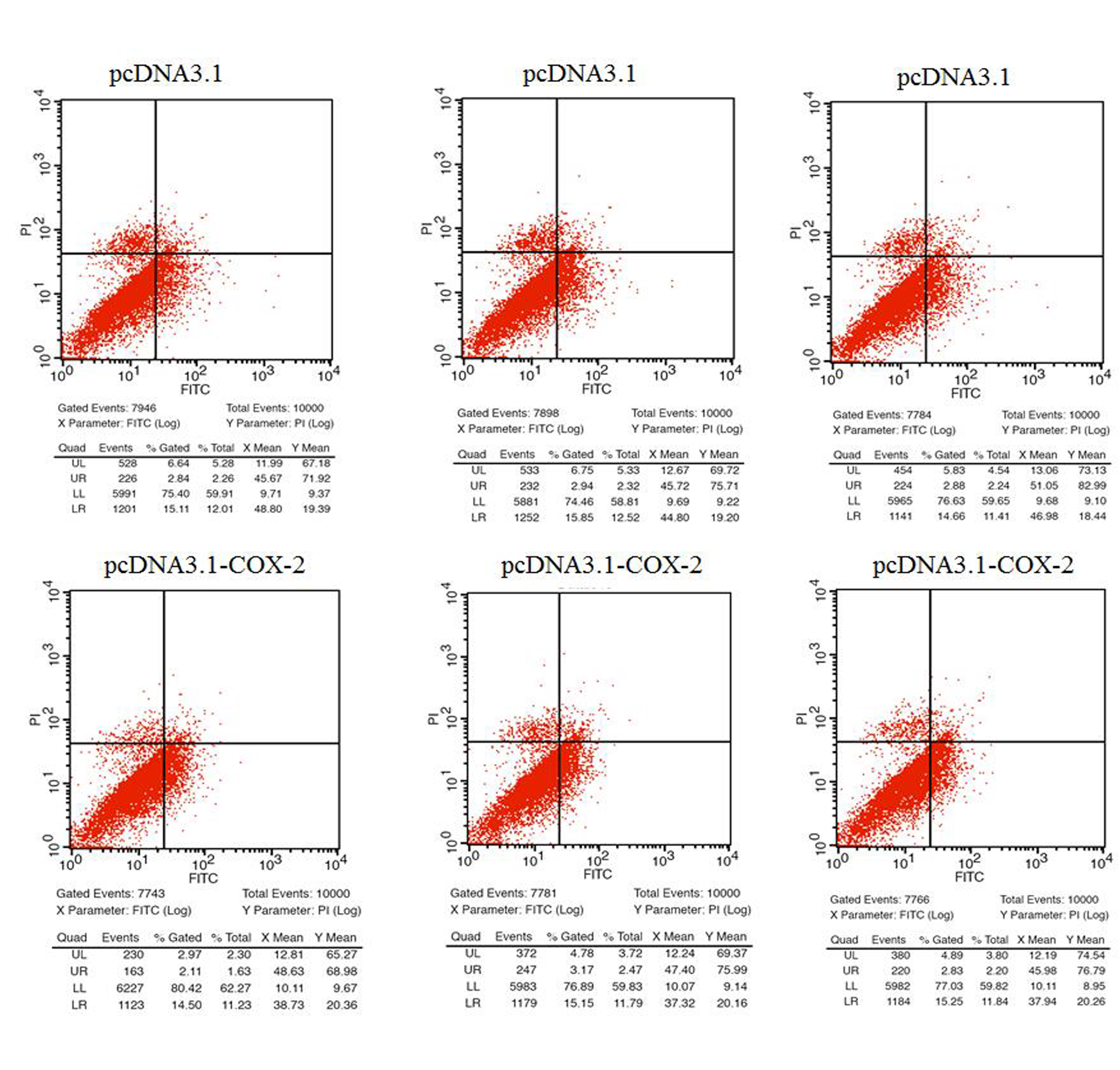


**Figure S9** Flow cytometry was used to detect mGC apoptosis after mGCs were transfected with *pcDNA3.1-COX-2* or *pcDNA3.1*

*
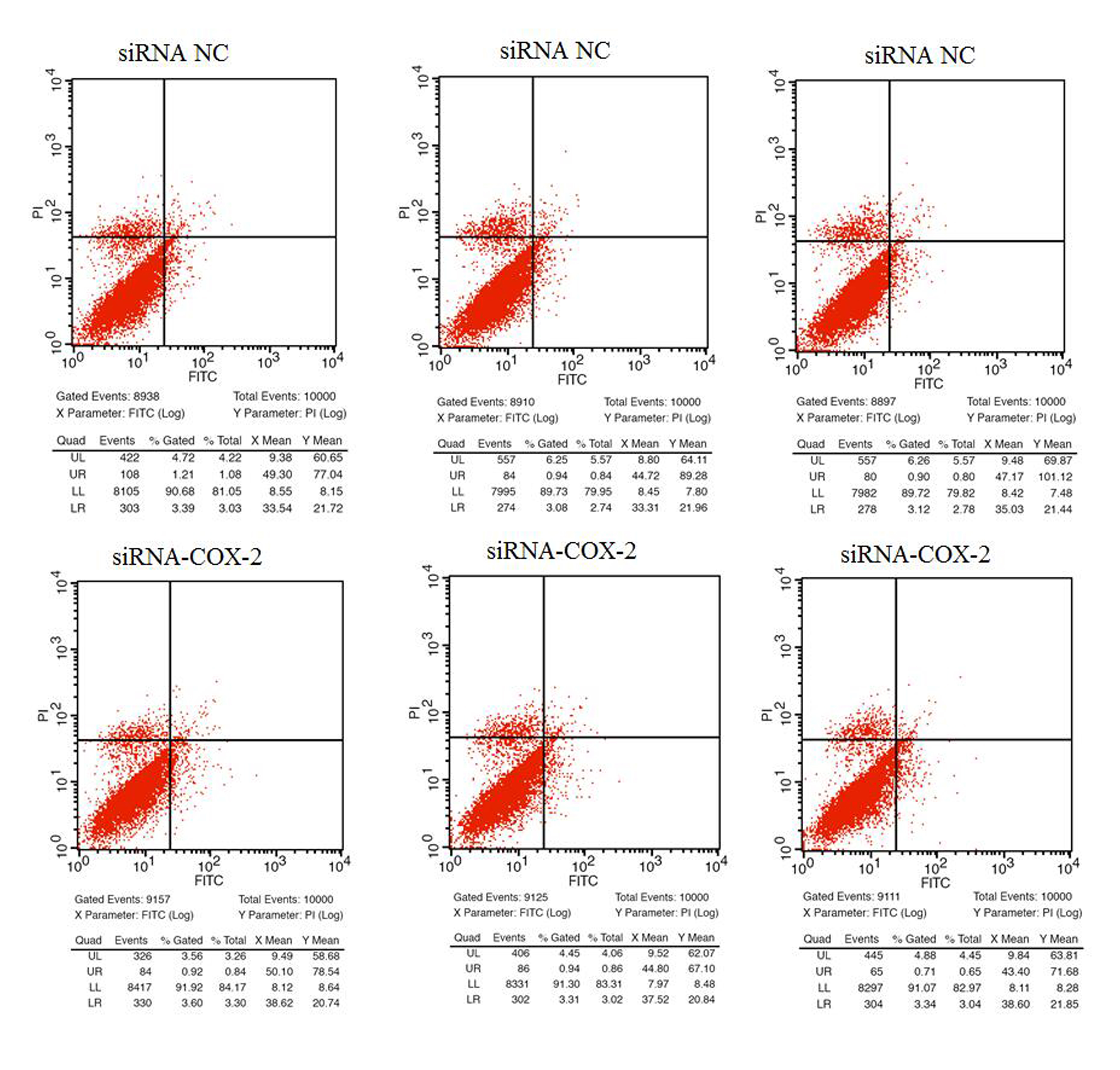
*

**Figure S10** Flow cytometry was used to detect mGC apoptosis after mGCs were transfected with siRNA-COX-2 or siRNA NC


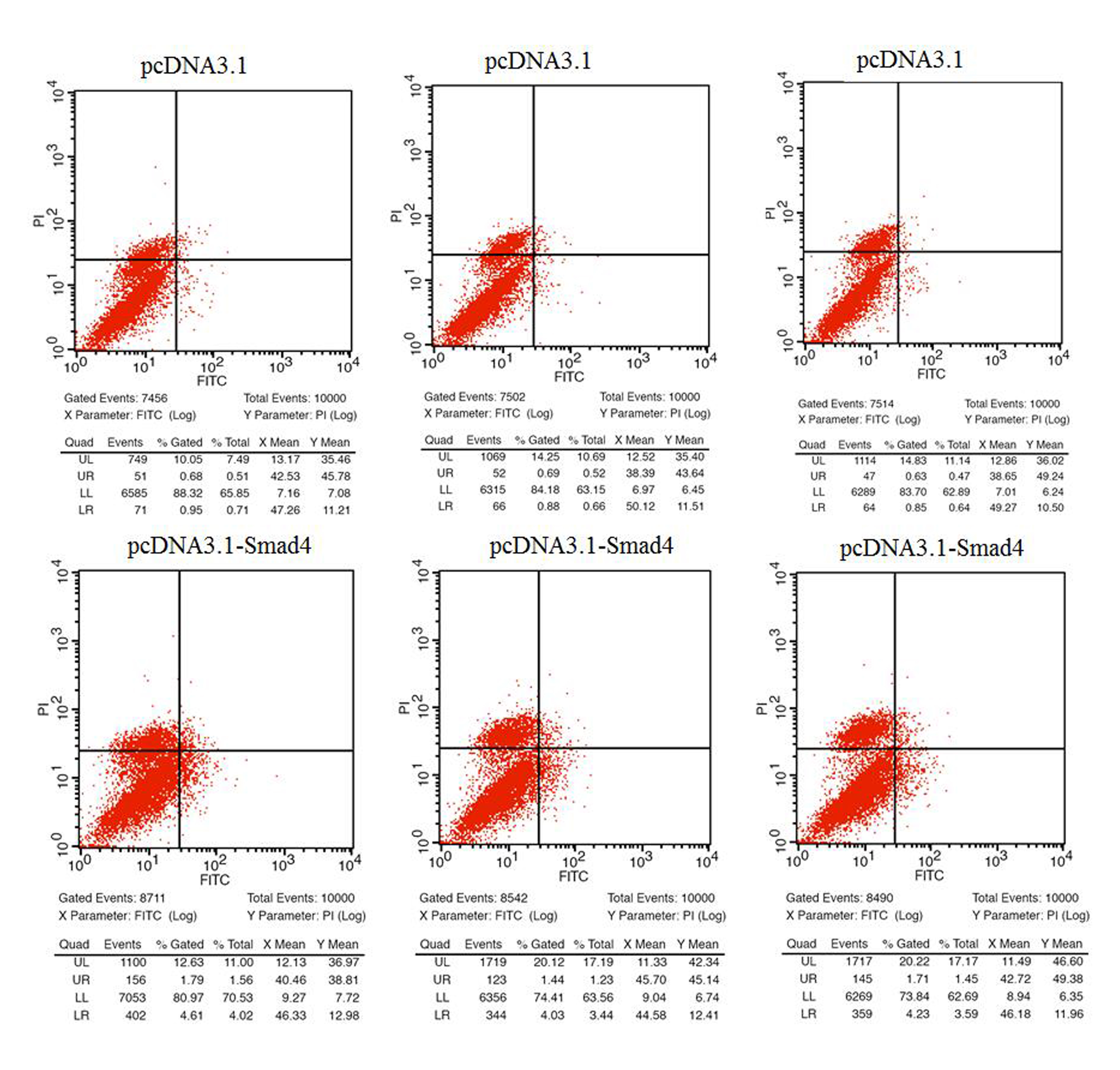


**Figure S11** Flow cytometry was used to detect mGC apoptosis after mGCs were transfected with *pcDNA3.1-Smad4* or *pcDNA3.1*

*
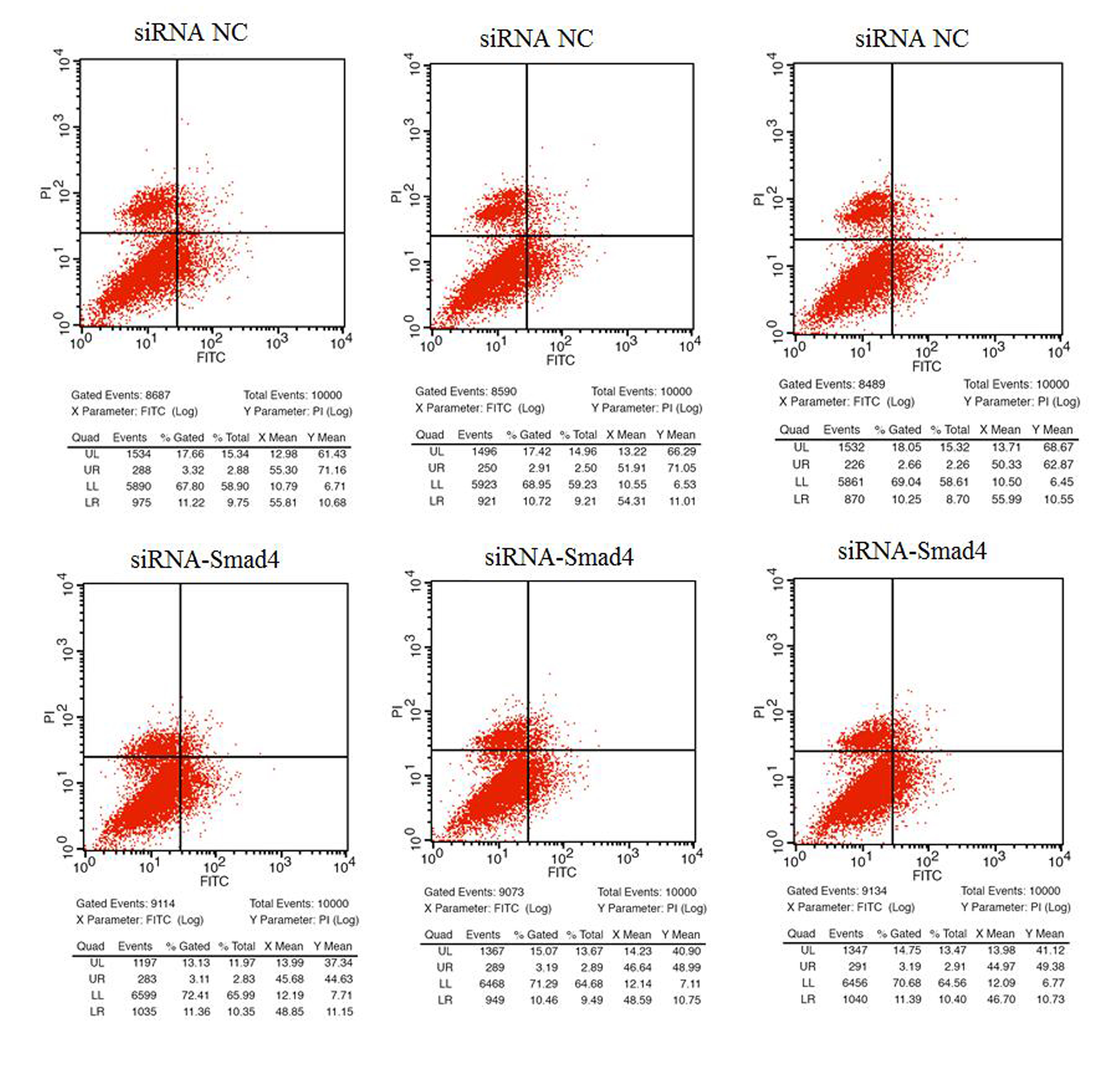
*

**Figure S12** Flow cytometry was used to detect mGC apoptosis after mGCs were transfected with siRNA-Smad4 or siRNA NC


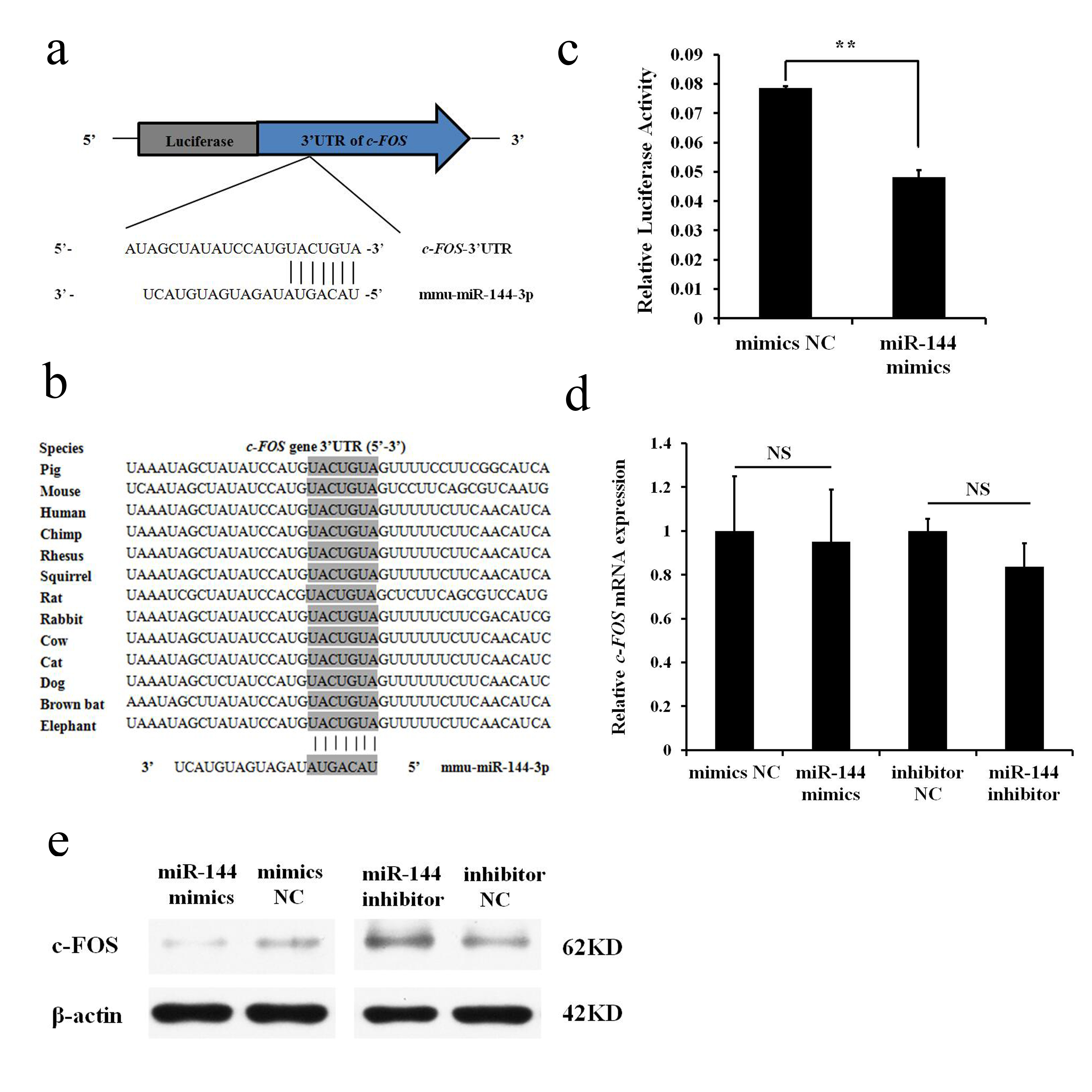


**Figure S13** Identification of *c-FOS* as a direct target of miR-144 in mGCs. (**a**) Binding sites for miR-144 in the 3’UTR of *c-FOS* predicted by TargetScan. (**b**) The miR-144 binding site sequences in the *c-FOS* 3’UTR in different species. (**c**) Luciferase activity was analyzed 24 h after mGCs were co-transfected with *pmiRGLO-c-FOS-3’UTR* and miR-144 mimics or mimics NC. (**d**) Endogenous *c-FOS* mRNA levels were detected 48 h after mGCs were transfected with miR-144 mimics, mimics NC, miR-144 inhibitor or inhibitor NC. (**e**) Western blot was used to detect endogenous c-FOS protein expression level 72 h after mGCs were transfected with miR-144 mimics, mimics NC, miR-144 inhibitor or inhibitor NC. The results are expressed as the mean ± S.E.M. (three independent replicates per group). ***P*<0.01, N.S. = non-significant


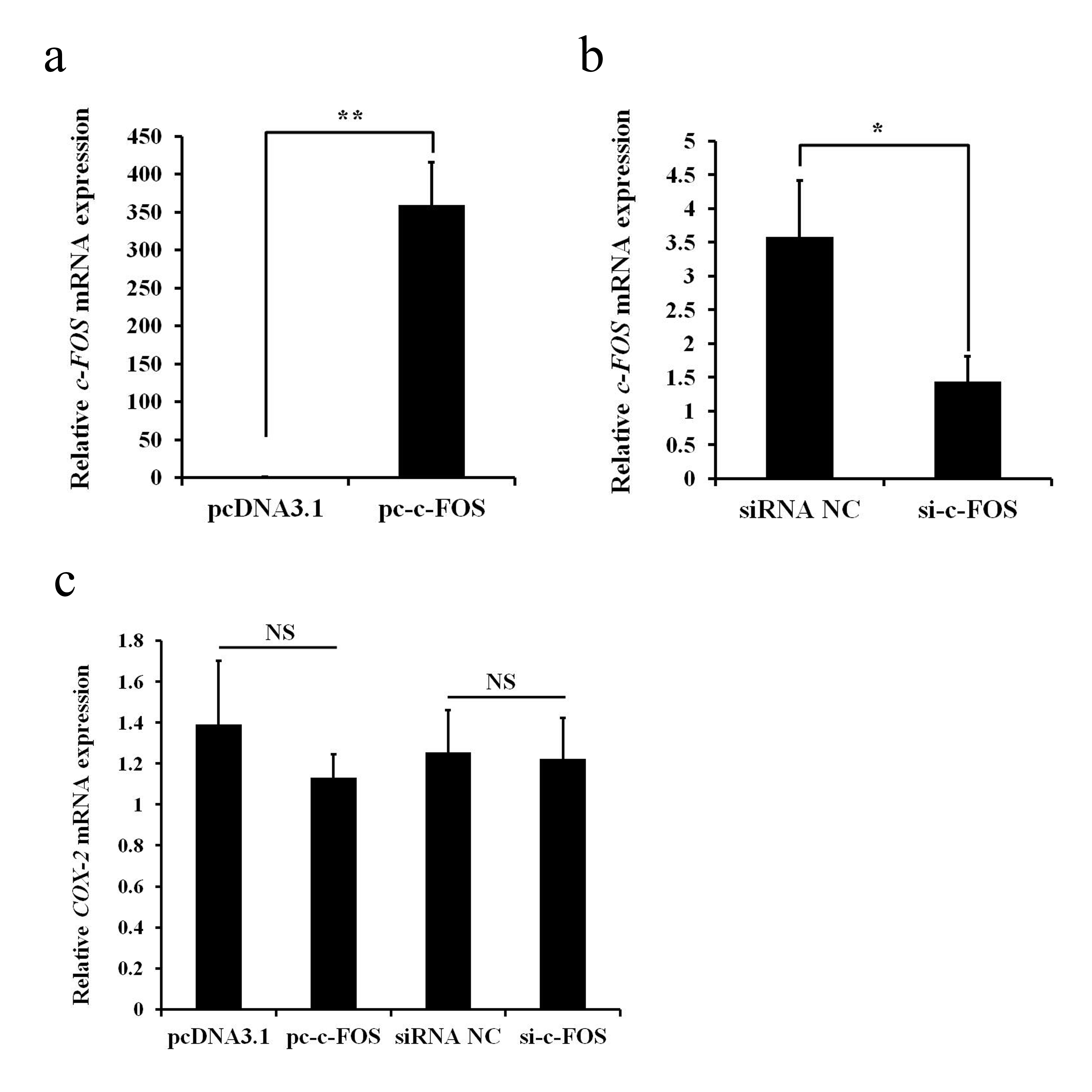


**Figure S14** *c-F0S* couldn’t regulate *COX-2* expression in mGCs. (**a**) The *pcDNA3.1-c-FOS* or *pcDNA3.1* was transfected into mGCs, and *c-FOS* mRNA expression level was measured by qRT-PCR. (**b**) The siRNA-c-FOS or siRNA NC was transfected into mGCs, and *c-FOS* mRNA expression level was measured by qRT-PCR. (**c**) Endogenous *COX-2* mRNA levels were detected 48 h after mGCs were transfected with *pcDNA3.1-c-FOS*, *pcDNA3.1*, siRNA-c-FOS or siRNA NC. The results are expressed as the mean ± S.E.M. (three independent replicates per group). **P*<0.05, ***P*<0.01, N.S. = non-significant
