# Supplementary material for: MicroRNA-144 is regulated by CP2 and decreases COX-2 expression and PGE2 production in mouse ovarian granulosa cells
Source: Cell Death Dis. 2017 Feb 9;8(2):e2597–. doi: 10.1038/cddis.2017.24 (PMC5386473; doi:10.1038/cddis.2017.24)
Supplement: Supplementary Table 2 [file cddis201724x3.doc]

Supplementary Table S2: Primer sequences

| Name Sequence (5’-3’) |
| --- |
| mmu-COX-2-CDS-PF CTAGCTAGCGCCACC CCTCTGCGATGCTCTTC  mmu-COX-2-CDS-PR CCCAAGCTT TTACAGCTCAGTTGAACGC  ssc-COX-2-qPCR-PF GGGTTGCTGGTGGTAGGAATC  ssc-COX-2-qPCR-PR GCATCTGGGCGAGGCTTT  mmu-COX-2-qPCR-PF TGCACTATGGTTACAAAAGCTGG  mmu-COX-2-qPCR-PR TCAGGAAGCTCCTTATTTC  ssc-COX-2-3’UTR-PF GGGTTTAAACTTTGCTGGA GAAGTAGGTT  ssc-COX-2-3’UTR-PR CCCTCGAGCAGAAAAATCGTCACAAGTATG  mmu-COX-2-3’UTR-PF GGGTTTAAACGTTCTGGGCAATAGGGATAA |
| mmu-COX-2-3’UTR-PR CCGCTCGAGTTGTTTGATTGTTCACACCAT  mmu-COX-2-3’UTR-mut-PF GCTGAGGTTGACTATATTACTG  mmu-COX-2-3’UTR-mut-PR CAGTAATATAGTCAACCTCAGC  mmu-Smad4-CDS-PF CTAGCTAGC ATGGACAATATGTCTATAACAA  mmu-Smad4-CDS-PR CCCAAGCTT TCAGTCTAAAGGCTGTGGGTCC  mmu-Smad4-qPCR-PF AGTAACGATGCCTGTCTGA  mmu-Smad4-qPCR-PR CACCTGAAGTCGTCCATC  mmu-Smad4-3’UTR-PF GGGTTTAAACCACTCTGCCTGCTGCTTC  mmu-Smad4-3’UTR-PR CCGCTCGAGACAAAGTCACCGCCAAGT  mmu-Smad4-3’UTR-mut-PF TATTCTGCAACTGATTTAACAT  mmu-Smad4-3’UTR-mut-PR ATGTTAAATCAGTT GCAGAATA  mmu-CP2-CDS-PF CGGGGTACCGCCACCGGTCGTGGTGGCTAAG |
| mmu-CP2-CDS-PR TGCTCTAGA GTGGGAAGTCCGTCAGT  mmu-CP2-qPCR-PF GCACTCGGCCAGCTGCCAGA  mmu-CP2-qPCR-PR TGGAGGGGGTGGTTCTGGCT  mmu-CP2-mut-PF GGGAAAACGTGTATATAGTTCGGGAGGGGCC  mmu-CP2-mut-PR GGCCCCTCCCGAACTATATACACGTTTTCCC  mmu-c-FOS-CDS-PF CTAGCTAGCGCCACCATGATGTTCTCGGGTTTCAA  mmu-c-FOS-CDS-PR CCCAAGCTTTCACAGGGCCAGCAGCGT  mmu-c-FOS-qPCR-PF AGACCGTGTCAGGAGGCA  mmu-c-FOS-qPCR-PR CCATCTTATTCCGTTCCCT  mmu-c-FOS-3’UTR-PF GGGTTTAAACTAACCTGGTGCTGGATTG  mmu-c-FOS-3’UTR-PR CCGCTCGAGATGAACATTGACGCTGAA  ssc-ACTB-qPCR-PF CCAGGTCATCACCATCGG  ssc-ACTB-qPCR-PR CCGTGTTGGCGTAGAGGT  mmu-ACTB-qPCR-PF GGCACCACACCTTCTACAATG  mmu-ACTB-qPCR-PR GGGGTGTTGAAGGTCTCAAAC  mmu-miR-144-D1-PF CTAGCTAGCCTTCGCTTATTTGAACTCC  mmu-miR-144-D2-PF CTAGCTAGCTGGGCTACATACAAAGTTCC |
| mmu-miR-144-D3-PF CTAGCTAGCCAGCCTGGTCTACAAAGTG  mmu-miR-144-D4-PF CTAGCTAGCGAGACCCAAACATATCAGGC |
| mmu-miR-144-D5-PF CTAGCTAGC ACTTAGAAGACGGGAGGC  mmu-miR-144-D6-PF CTAGCTAGC GGAAGCCAGCAGGCAAAG  mmu-miR-144-D7-PF CTAGCTAGC AGCTGCTTGAGTGAGAAGAG  mmu-miR-144-D8-PF CTAGCTAGC CAGAGGATTCCCTGGACG  mmu-miR-144-D9-PF CTAGCTAGC CGTTTCTGCCTGTAACTCTG  mmu-miR-144-D-PR CCAAGCTT TATCCCAGCCAAGGTCC  ChIP –PF GCCAGCAGGCAAAGAGTT  ChIP –PR GAGCCTCGTCCAGGGAAT  mmu-miR-144-Loop GTCGTATCCAGTGCAGGGTCCGAGGTATTCGCAC  TGGATACGACAGTACATC  mmu-miR-144-PF GCCCCTACAGTATAGATGATGTA  mmu-miR-144-PR GTGCAGGGTCCGAGGT  mmu-miR-451-Loop CTCAACTGGTGTCGTGGAGTCGGCAATTCAGTTG  AGAACTCAGT  mmu-miR-451-PF TCGGCAGGAAACCGTTACCATT  mmu-miR-451-PR TGCAGGTCAACTGGTGTCGT  mmu-U6-Loop CTCAACTGGTGTCGTGGAGTCGGCAATTCAGTTG  AGAAAAATATGGAACGCT  mmu-U6-PF CTGGTAGGGTGCTCGCTTCGGCAG  mmu-U6-PR CAACTGGTGTCGTGGAGTCGGC  ssc-let-7a-Loop CTCAACTGGTGTCGTGGAGTCGGCAATTCAGTT  GAGAACTATAC  ssc-let-7a-PF CTGGTAGGTGAGGTAGTAGG  ssc-let-7a-PR GGTGTCGTGGAGTCGGCAAT  ssc-miR-125a-Loop CTCAACTGGTGTCGTGGAGTCGGCAATTCAGTT  GAGCACAGGTT  ssc-miR-125a-PF TCCCTGAGACCCTTTAACCT  ssc-miR-125a-PR TGTCGTGGAGTCGGCAAT  ssc-miR-144-Loop GTCGTATCCAGTGCAGGGTCCG AGGTATTCGCA  CTGGATACGACAGTACATC  ssc-miR-144-PF GCCCCTACAGTATAGATGATGTA  ssc-miR-144-PR GTGCAGGGTCCGAGGT  ssc-miR-3613-5p-Loop CTCAACTGGTGTCGTGGAGTCGGCAATTCAGTT  GAGTAACAAAA  ssc-miR-3613-5p-PF TGGTGTCGTGGAGTCGGC  ssc-miR-3613-5p-PR GCAGGGTGTTCTACTTTTATTTTTG  ssc-miR-331*-Loop CTCAACTGGTGTCGTGGAGTCGGCAATTCAGTT  GAGAACAAACC  ssc-miR-331*-PF TTTGTTTGGGTTTGTTCTC  ssc-miR-331*-PR TGGTGTCGTGGACTCGGC  ssc-miR-2423-Loop CTCAACTGGTGTCGTGGAGTCGGCAATTCAGTT  GAGAGAGAAAAC  ssc-miR-2423-PF GCAGGGTTTGTGTTTTGTTTTCTCT  ssc-miR-2423-PR TCAACTGGTGTCGTGGACTCGGC  ssc-miR-4028-3p-Loop CTCAACTGGTGTCGTGGAGTCGGCAATTCAGTT  GAGAAAAATAAC  ssc-miR-4028-3p-PF TTTTTTTGTTCTTGTTGTTGCTCTC  ssc-miR-4028-3p-PR TGGTGTCGTGGACTCGGC  ssc-n-miR-1-Loop CTCAACTGGTGTCGTGGAGTCGGCAATTCAGTT  GAGCCAGAGGT  ssc-n-miR-1-PF TCCCTCTGTGAACTAGAAACCT  ssc-n-miR-1-PR TGTCGTGGAGTCGGCAAT  ssc-n-miR-2-Loop CTCAACTGGTGTCGTGGAGTCGGCAATTCAGT  TGAGGACTGCAC  ssc-n-miR-2-PF CGTCCCTGCGTGGCTTCT  ssc-n-miR-2-PR GCTGGTGTCGTGGAGTCGG  ssc-n-miR-3-Loop CTCAACTGGTGTCGTGGAGTCGGCAATTCAGT  TGAGTCACTTCA  ssc-n-miR-3-PF TCGGCAGGTTTGTTGGCTCCTC  ssc-n-miR-3-PR TCAACTGGTGTCGTGGAGTCGGCAA  ssc-U6-PF GCTTCGGCAGCACATATACT  ssc-U6-PR TTCACGAATTTGCGTGTCAT |

Note: PF was upstream primer. PR was downstream primer. The part highlighted with grey was enzyme site induced.
